# A Neuronal Network Model for Pitch Selectivity and Representation

**DOI:** 10.3389/fncom.2016.00057

**Published:** 2016-06-16

**Authors:** Chengcheng Huang, John Rinzel

**Affiliations:** ^1^Department of Mathematics, Courant Institute of Mathematical Sciences, New York UniversityNew York, NY, USA; ^2^Department of Mathematics, University of PittsburghPittsburgh, PA, USA; ^3^Center for Neural Science, New York UniversityNew York, NY, USA

**Keywords:** pitch, slope-detector, missing fundamental, inharmonics, alternating phase, Schroeder phase, iterated-ripple-noise

## Abstract

Pitch is a perceptual correlate of periodicity. Sounds with distinct spectra can elicit the same pitch. Despite the importance of pitch perception, understanding the cellular mechanism of pitch perception is still a major challenge and a mechanistic model of pitch is lacking. A multi-stage neuronal network model is developed for pitch frequency estimation using biophysically-based, high-resolution coincidence detector neurons. The neuronal units respond only to highly coincident input among convergent auditory nerve fibers across frequency channels. Their selectivity for only very fast rising slopes of convergent input enables these slope-detectors to distinguish the most prominent coincidences in multi-peaked input time courses. Pitch can then be estimated from the first-order interspike intervals of the slope-detectors. The regular firing pattern of the slope-detector neurons are similar for sounds sharing the same pitch despite the distinct timbres. The decoded pitch strengths also correlate well with the salience of pitch perception as reported by human listeners. Therefore, our model can serve as a neural representation for pitch. Our model performs successfully in estimating the pitch of missing fundamental complexes and reproducing the pitch variation with respect to the frequency shift of inharmonic complexes. It also accounts for the phase sensitivity of pitch perception in the cases of Schroeder phase, alternating phase and random phase relationships. Moreover, our model can also be applied to stochastic sound stimuli, iterated-ripple-noise, and account for their multiple pitch perceptions.

## 1. Introduction

Pitch is a perceptual correlate of periodicity (Oxenham, 2012). Operationally, the pitch of a sound can be measured by matching it to a pure tone (Hartmann, 1997). Sounds with the same repetition rate generally share the same pitch. The spectra of the sounds, however, can be distinct, which results in different timbre. The equivalence of pitch is the basis of how we recognize the same piece of music played by different instruments. Pitch is also an important cue to group together harmonic frequencies that often arise from the same vibration source and helps segregate sound sources.

The pitch of a pure tone is its tone frequency, which can be simply encoded as a place code in the tonotopy. The pitch frequency of a complex tone, however, may not be present in the spectrum of the tone. For example, the pitch of a harmonic complex is its fundamental frequency (F0), the maximum common divisor of its frequency components, regardless of whether the F0 component is present in the sound or not. This property suggests that the pitch of a sound is encoded as an integrated feature of all frequency components. The perceptual phenomenon of identifying F0 as a sound's pitch when the F0 component is absent is called the “pitch of the missing fundamental (MF)” and has been a benchmark in attempts to seek neural correlates of pitch (Schwarz and Tomlinson, 1990; Fishman et al., 1998; Bendor and Wang, 2005). Bendor and Wang (2005) found pitch-selective neurons in the rostral region of auditory cortex that respond to MF harmonics even when the harmonic frequencies are outside the neuron's receptive field.

Computational models in the pitch literature generally fall under one of the two categories, spectral models and temporal models (for a review, see de Cheveigné, 2005). Spectral pitch models assume the existence of spectral templates of harmonics that are associated with their F0s (Goldstein, 1973; Wightman, 1973a; Terhardt, 1979). The pitch of an MF complex can then be decoded by finding the best matched template. Shamma and Klein (2000) suggests that such templates can be generated by across-frequency coincidence detection. The spectral models work well for the pitch of resolved harmonics, harmonics that are well separated in the cochlea, but not for the pitch of unresolved harmonics.

On the other hand, temporal models utilize phase-locking properties of the auditory nerve (AN) responses (Licklider, 1951; Lyon, 1984; Meddis and Hewitt, 1991a,b; Patterson, 1994; de Cheveigné, 1998; Balaguer-Ballester et al., 2008). Many temporal models use autocorrelation (AC) functions of spike trains to extract periodicity of AN responses across frequency channels. Then the AC functions over all frequency channels are summed to extract the common periodicity across frequencies as the pitch-related periodicity. Those temporal models are supported by electrophysiological recordings from the AN fibers in cat (Cariani and Delgutte, 1996a,b), for which it was found that the largest peaks in the pooled AC histograms of all AN fibers are related to pitch for a variety of pitch phenomena. However, a biophysical implementation of a delay line to compute an AC function remains an open problem (Shamma et al., 1989; de Cheveigné and Pressnitzer, 2006).

Neurons of the cochlear nucleus have shown enhancement of pitch representation in their first-order inter-spike interval (ISI) distributions compared to the AN fibers (Joris et al., 1994a,b; Rhode, 1995, 1998). Onset type units can strongly phase-lock at both the F0 of harmonic complexes and the modulation rate of amplitude-modulated tones. Neurons with sharp onset responses followed by a short refractory period also show significant improvement in envelope coding compared to AN fibers (Rhode, 1995, 1998). Neurons in those categories, such as bushy cells and octopus cells, have high temporal precision in phase-locking and in their transient onset responses. Based on *in vitro* electrophysiology, Rothman and Manis (2003) characterized the biophysical profile and developed models of phasic firing neurons in the ventral cochlear nucleus. Such neurons and models are good at monaural coincidence detection. We use a reduced version of such models (Meng et al., 2012) as slope-detectors to detect coincidence among AN fibers and to encode pitch information. Our slope-detectors are exceptional at detecting temporal coincidence. They differ from the simple coincidence detector units used by previous temporal models, based on multiplicative operations of two spike trains, in that our slope-detectors are sensitive to the rising slope of input and do not fire repetitively for steady input. The phasic property enables the slope-detectors to typically fire one spike per cycle when receiving half-wave rectified sinusoidal inputs (Meng et al., 2012) and have a band-pass frequency tuning.

Our computational model encodes pitch by detecting coincidence among frequency channels using biophysical slope-detectors. The model consists of three stages: (1) transduction of sound to neural activity in individual frequency channels (AN model), (2) convergence of channel outputs to a neuronal network of slope-detectors (SDs) that compute periodicity and (3) pitch estimation from the first-order ISI distributions of the SD units. Each SD unit receives a broad range of inputs from the AN fibers and phase-locks at the F0 of the MF harmonic complexes. Pitch frequency can then be estimated by choosing the most frequently occurring first-order ISI in the pooled histograms of all SDs. We test our model by demonstrating its performance in several pitch perception scenarios. First, the model can detect the F0 of the MF complexes and the decoded pitch strength decreases with the lowest harmonic number. Second, the estimated pitch varies with the shift frequency of inharmonic complexes. Third, the model can account for the phase sensitivity and insensitivity of pitch in examples of the Schroeder phase, the alternating phase and random phase relationships. Lastly, our model can also estimate the pitch of iterated-ripple-noise stimuli generated by delay-add or delay-subtract operations.

## 2. Materials and methods

### 2.1. Model structure

**Figure 1:**
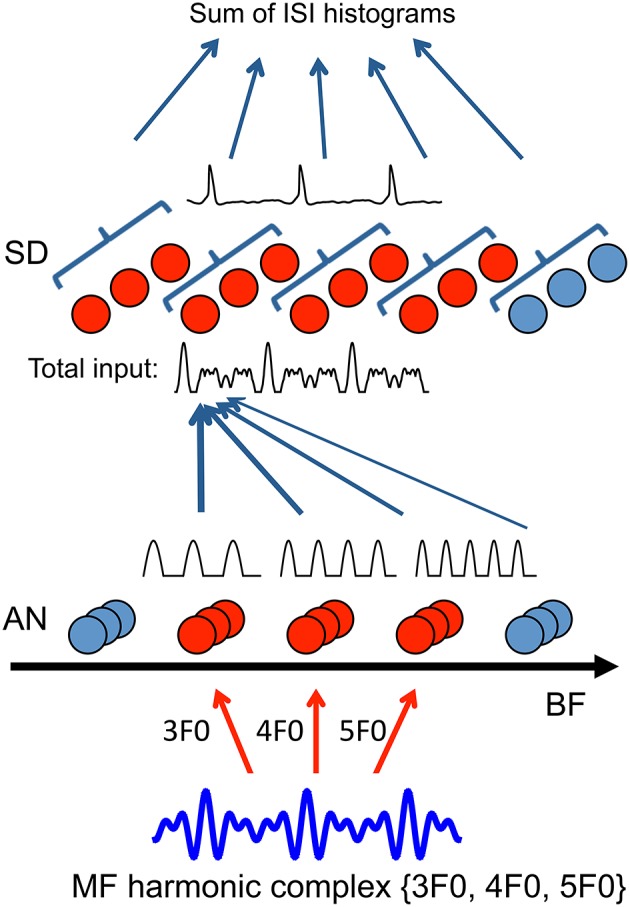
**Model schematic (details see Methods)**. Auditory nerve (AN) fibers across frequency channels (20 AN fibers per BF channel) converge to a layer of tonotopically organized, uncoupled slope-detector (SD) units with one-sided footprint (σ = 2 octaves, Equation 7). First-order ISIs of the spike trains from SD units at the same center frequency (CF) are pooled together to generate an ISI histogram (10 SDs per CF). Pitch is decoded by choosing the largest peak in the sum of ISI histograms across CF. To illustrate the model mechanism, AN inputs are approximated as rectified sine-waves of corresponding BFs and only channels of the frequencies that are present in the sound are activated. When a missing fundamental with frequency components {3F0, 4F0, 5F0} is delivered, AN fibers from BF channels 3F0, 4F0, and 5F0 are activated (red). Inputs traces (rectified sine-waves) within one cycle of 1/F0 are shown above the AN units. Each SD unit (red) receives a weighted sum of AN inputs from frequency channels above the SD unit's CF (total input shows one example trace of 3 cycles). SD units can phase-lock at the fundamental frequency F0 (one example of membrane potential trace shown above SD units), resulting in a peak at 1/F0 in their ISI histogram. Responses of the full model with a detailed AN model is shown in Figure 2.

The model consists of three stages: transduction of sound to neural activity in individual frequency channels (AN model), convergence of channels to compute periodicity by slope-detectors (SD) and pitch frequency estimation (Figure 1). The sound stimulus is first processed by an AN model, which transforms the sound into spike trains from different frequency channels. The AN fibers across frequency channels converge to uncoupled SD units. The SD units can detect coincidence among the spike trains from different frequency channels and phase-lock at the fundamental frequency (F0) of the sound. The model is strictly feedforward without explicit recurrent synapses before or at the stage of the SD units. To estimate pitch from the temporal patterns of the SD spike trains, we choose as the decoded pitch the inverse of the most frequently occurring ISI of all SDs.

#### 2.1.1. The auditory nerve model

We generate AN spike trains using the Matlab Auditory Periphery (MAP) software package (MAP1-14h) developed by Meddis et al. (2013) and Meddis and O'Mard (1997) (the package can be downloaded from http://www.essexpsychology.macmate.me/resources/software/MAP1_14h-public.zip). This model consists of a cascade of six stages from the auditory periphery up to the auditory nerve: (1) middle ear filtering; (2) basilar membrane modeled by dual-resonance nonlinear filters; (3) inner hair cell receptor potential; (4) inner hair cell presynaptic calcium currents; (5) transmitter release events at the inner hair cell to AN synapse; and (6) AN spiking response including refractory effects. Spike trains are generated independently for each AN fiber of specified best frequency (BF), which is the frequency generating the greatest response near hearing threshold.

#### 2.1.2. Slope-detector model

**Figure 2:**
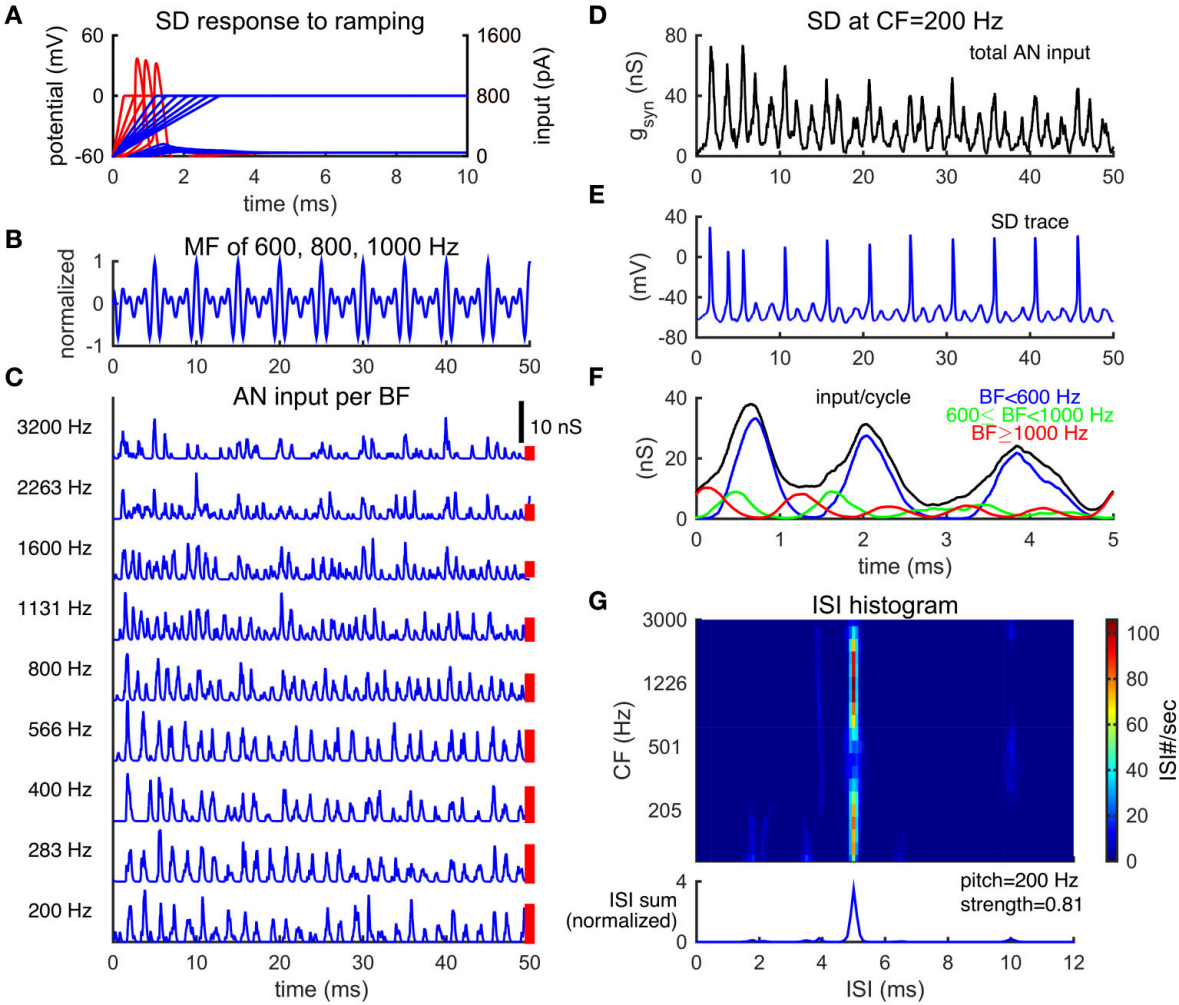
**Slope-detector units can phase-lock at the fundamental frequency of a missing fundamental complex**. **(A)** Response of a SD unit to ramping input. An SD unit only fires once when input rises fast enough (red). No action potential is generated for slowly ramped input (blue). Left ordinate is for membrane potential (mV) and right ordinate is for input current (pA). **(B)** Normalized waveform of an MF complex of harmonics {600, 800, 1000} Hz (F0 = 200 Hz) in cosine phase. **(C)** AN inputs, time courses of post-synaptic conductance, from representative BF channels in response to the MF tone shown in **(B)**. The corresponding BFs are shown on the left. Each trace is a combination of 20 AN fibers and is not weighted by the footprint function ω (Equation 7). AN spikes are generated using Matlab Auditory Periphery package (MAP1-14h) developed by Meddis et al. (2013). **(D)** Total conductance input, *g*_*syn*_ (Equation 4), to an SD unit at center frequency (CF) 200 Hz from AN fibers shown in **(A)**. This SD unit receives inputs from AN channels of BF 200 Hz and above, weighted by the footprint function ω (Equation 7) with width σ = 2 octaves. The weight on each AN input across BF is indicated by the height of a red bar to the right of the corresponding trace in **(C)** (arbitrary unit). **(E)** The SD unit at CF = 200 Hz phase-locks at the fundamental frequency F0. **(F)** The time-averaged input over period 1/F0 for the SD unit at CF = 200 Hz, same trial as that shown in **(D)**. The summed input from AN channels of BF lower than 600 Hz (blue) dominates the total input. The summed input from AN channels of BF from 600 Hz to 1000 Hz (green) and that from BF higher than 1000 Hz (red) are modulated at 800 and 1000 Hz, respectively. The total input (black) shows the steepest rising portion at the first peak and two succeeding shallower bumps of inputs per cycle. Results are averaged over one trial of stimulus duration 100 ms (20 cycles). **(G)** ISI histograms of SD units across CF show peaks at 5 ms (1/F0) (color plot). The summed ISI histogram over CF, therefore, has a peak at 5 ms (bottom, area under the curve is normalized to 1). The decoded pitch to this MF complex shown in B is 200 Hz, same as its F0. Pitch strength is high (0.81), indicating salient pitch perception.

The second stage consists of uncoupled SD units that are temporally precise coincidence detectors, sensitive to the rising slope of input. An SD unit displays a “phasic” firing property, meaning that it only fires once when an input rises fast enough and does not fire repeatedly for slow or steady inputs (Meng et al., 2012) (Figure 2A). Such behavior is generally due to a dynamic subthreshold negative feedback mechanism. Rothman and Manis (2003) found that a slowly-inactivating, low-threshold potassium current (*I*_*KLT*_) was mainly responsible for the phasic firing and developed a biophysical cellular model for neurons from the auditory brain stem where temporal processing is important. Here, for each SD unit, we use a reduced cellular model (Meng et al., 2012) that successfully captures the main features of the model of Rothman and Manis (2003). The differential equations for each SD are

(1) $$\begin{array}{l} C_{m}\frac{dV}{dt}=-2[{\bar{g}}_{Na}ab^{-1}m_{\infty }^{3}(V)U(V-E_{Na})\\ \;+\;{\bar{g}}_{KLT}{\left(a-aU\right)}^{4}z_{0}(V-E_{K})\\ \;+{\bar{g}}_{KHT}(0.85n_{0}^{2}+0.15p_{0})(V-E_{K})\\ \;+\;{\bar{g}}_{h}r_{0}(V-E_{h})+{\bar{g}}_{L}(V-E_{L})]+I(t)\\ \;\frac{dU}{dt}=3\frac{U_{\infty }(V)-U}{\tau_{U}(V)}. \end{array}$$

The steady-state function *U*_∞_(*V*) is given by

(2) $$\begin{array}{l} U_{\infty }\left(V\right)=\frac{b\left[h_{\infty }\left(V\right)+b\left(a-w_{\infty }\left(V\right)\right)\right]}{a\left(1+b^{2}\right)}, \end{array}$$

where *a* = 0.9, *b* = (*a* − *w*_0_)/*h*_0_ and τ_*U*_(*V*) = min(τ_*w*_(*V*), τ_*h*_(*V*)). The variable *U* combines two negative-feedback gating variables that have similar time scales: activation (*w*) of *I*_*KLT*_ and inactivation (*h*) of *I*_*Na*_ (Meng et al., 2012). The constants, *z*_0_, *n*_0_, *p*_0_ and *r*_0_ correspond to “freezing” some gating variables to their resting values. The expressions for *h*_∞_(*V*), *w*_∞_(*V*), τ_*w*_(*V*) and τ_*h*_(*V*) are obtained experimentally from voltage-clamp responses and are given in Rothman and Manis (2003, for parameter values and functions see Table I, Type II). *I*(*t*) is the total synaptic current each SD unit receives from AN fibers (Equation 3). A spike is recorded when the membrane potential, *V*, crosses our criterion level, −10 mV. Numerical integration is implemented with Matlab function *ode45*, which uses use an explicit Runge-Kutta method of 4th order accuracy, with error tolerance 10^−5^.

#### 2.1.3. Connectivity from AN to SD

The total synaptic current *I*(*t*) that each SD unit at center frequency (CF) *f*_*SD*_ receives from the AN fibers is

(3) $$\begin{array}{l} I\left(t\right)=g_{syn}\left(t\right)\left(V-V_{E}\right) \end{array}$$

where *V*_*E*_ = 0 mV is the reversal potential of excitatory synapses. Here the CF of an SD unit refers to the frequency of AN fibers from which it receives the largest input. The total synaptic conductance from AN, *g*_*syn*_(*t*), is

(4) $$\begin{array}{l} g_{syn}\left(f_{SD},t\right)=\underset{f_{AN}}{\sum }\omega \left(f_{AN},f_{SD}\right)g_{AN}\left(f_{AN},t\right). \end{array}$$

*g*_*AN*_(*f*_*AN*_, *t*) is the synaptic conductance from AN fibers of BF *f*_*AN*_,

(5) $$\begin{array}{l} g_{AN}\left(f_{AN},t\right)=\Sigma_{i}{ḡ}_{E}\eta \left(t-t_{i}\right), \end{array}$$

where *t*_*i*_'s are the spike times of the AN fibers and η(*t*) is the post-synaptic potential generated by a single AN spike,

(6) $$\begin{array}{l} \eta \left(t\right)=\frac{t}{\tau_{E}}e^{\left(-t/\tau_{E}\right)}, \end{array}$$

which is an alpha function with a maximum 1 at *t* = τ_*E*_. We use τ_*E*_ = 0.07 ms and *g*_*E*_ = 1.5 nS, unless otherwise stated.

The footprint from AN to SD, ω(*f*_*AN*_, *f*_*SD*_), is a one-sided Gaussian of width σ = 2 octaves (Equation 7). Hence each SD unit can integrate information and detect coincidence from the AN fibers across BF channels. Use of a symmetric footprint would lead to a broader CF range of SDs in response to sound, but it would not change the results qualitatively.

(7) $$\begin{array}{l} \omega (f_{AN},f_{SD})=\{\begin{array}{ll} \exp(-\frac{{\left|\log(f_{AN})-\log(f_{SD})\right|}^{2}}{\sigma^{2}}), & f_{AN}⩾f_{SD}\\ 0, & f_{AN}<f_{SD} \end{array} \end{array}$$

There are 20 CF sites for SD units, equally spaced in logarithmic scale from 100 to 3000 Hz, with 10 uncoupled SD units per CF site. Each SD unit receives 20 independent AN fibers of high spontaneous rate per BF channel. In the implementation of the MAP program, there are 29 AN channels with BF equally-spaced in logarithmic scale from 50 to 6400 Hz, which is approximately 4 AN channels per octave, unless otherwise noted. Since the width of the footprint from AN to SD is 2 octaves (Equation 7), each SD unit receives AN input from approximately 8 BF sites with 20 AN fibers per site.

#### 2.1.4. Pitch frequency estimation

Pitch is decoded by choosing the largest peak in the pooled first-order ISI histograms of all SD units. The ISIs from the SD units at the same CF site are pooled together to generate an ISI histogram with a bin width of 0.1 ms. To estimate an overall pitch, the ISI histograms of all CF are summed and the ISI of the highest peak is chosen as the inverse of the decoded pitch (possible neuronal computation see Discussion). The strength of pitch is computed by dividing the number of ISIs near the highest peak (between the two nearest dips) by the sum of total ISIs. A similar definition was used by Cariani and Delgutte (1996a,b) to estimate pitch salience from all-order interval histograms of AN spike trains. We use vector strength (Equation 8) to quantify how well a spike train ${\left\{t_{i}\right\}}_{i=1}^{N}$ is phase-locked with respect to period *T*.

(8) $$\begin{array}{l} vs=\frac{1}{N}\sqrt{\Sigma_{i=1}^{N}\cos^{2}\left(2\pi t_{i}/T\right)+\Sigma_{i=1}^{N}\sin^{2}\left(2\pi t_{i}/T\right)} \end{array}$$

### 2.2. Auditory stimuli

Audio files of some representative sound stimuli can be found in the Supplementary Material.

#### 2.2.1. Harmonic complex

In a harmonic complex, frequency components are integer multiples of its fundamental frequency F0. The harmonic number index *n* corresponds to a frequency *f*_*n*_ = *nF*0. The general waveform equation is

(9) $$\begin{array}{l} s\left(t\right)=\overset{N}{\underset{n=1}{\sum }}\cos\left(2\pi f_{n}t+\phi_{n}\right) \end{array}$$

A harmonic complex is called a missing fundamental (MF) harmonic complex when it does not contain the F0 component (a waveform example see Figure 2B). We choose ϕ_*n*_ = 0 for cosine phase and ϕ_*n*_ = π/2 for sine phase. Sound duration is 100 ms with a 5 ms cosine ramp at onset and offset, unless otherwise noted. A silence of 50 ms is added before and after each stimulus in the implementation of the MAP program. The overall sound level is 65 dB. The sampling rate is 50 kHz.

#### 2.2.2. Inharmonic complex

An inharmonic complex is generated by shifting the frequency components of a harmonic complex by the same amount Δ*f*. The inharmonic complexes used in this paper contain frequencies *nF*0 + Δ*f* in cosine phase, where F0 = 100 Hz and *n* = 1, 2, …, 6.

#### 2.2.3. Schroeder phase complex

Schroeder phase is a specially designed phase relationship (Equation 10) for harmonic complexes to create a relatively flat temporal envelope (Schroeder, 1970).

(10) $$\begin{array}{l} \phi_{n}=c\pi n\left(n+1\right)/N, \end{array}$$

where *N* is the total number of harmonics. A positive Schroeder phase (*m*_+_) complex has *c* = 1 (**Figure 6A1**) and a negative Schroeder phase (*m*_−_) complex has *c* = −1 (**Figure 6A2**). In this paper, we use F0 = 100 Hz and harmonic number 2–50.

#### 2.2.4. Alternating phase harmonics

An alternating phase harmonics sound (ALT) is a harmonic complex with cosine phase (ϕ_*n*_ = 0) when *n* is even and sine phase (ϕ_*n*_ = π/2) when *n* is odd. The stimuli were generated by filtering a complex of 80 harmonics (F0 = 125 Hz) using a Butterworth filter in one of the three frequency regions (**Figure 7A1**): (1) LOW, 125–625 Hz; (2) MID, 1375–1875 Hz; (3) HIGH, 3900–5400 Hz (Shackleton and Carlyon, 1994). Overall sound level is 50 dB. To fully represent the high frequency region, 37 BF channels from 50 to 25600 Hz are used in the MAP program and 40 CF sites from 100 to 10000 Hz are used for the SD units. The unitary synaptic strength is *g*_*E*_ = 3 nS.

#### 2.2.5. Iterated ripple noise (IRN):

An IRN stimulus is generated by a cascade of delay and add operations of white noise (Yost et al., 1998). The procedures are the following: (1) create a segment of white noise *X*(*t*) and make a copy *X*_1_(*t*) = *X*(*t*). (2) Delay *X*(*t*) by *d* ms and add to the copy of the original noise with a gain factor *g*, obtaining the next iterate *X*(*t*) = *X*_1_(*t*) + *gX*(*t* − *d*). This is considered as one iteration. (3) Repeat step 2 to obtain *n* iterations. This procedure creates spectral ripple and temporal regularity in the noise. In this paper, we use *d* = 4 ms, *g* = 1 (addition) or *g* = −1 (subtraction) and *n* = 2, 4, 8. The addition and subtraction operations give distinct spectral peaks, yet very similar temporal envelopes (**Figures 9A,B**). The sound has duration of 300 ms with 20 ms ramp on and off and overall level of 70 dB. Parameter values are similar to that in Yost et al. (1998).

## 3. Results

We apply our model to a range of pitch-evoking sound stimuli which have been commonly used in the psychoacoustic literature of pitch and to assess pitch models. The pitch of a sound is related to both its spectral content, such as the fundamental frequency of a harmonic complex, and its waveform envelope. Many complex sound stimuli have been designed to dissect these two cues of pitch and to elucidate the principle mechanisms of pitch. We first illustrate the model's mechanism using the missing fundamental (MF) harmonic complex. We compare the tuning of pitch frequency for MF complexes and pure tones (Section 3.1). When the harmonics of F0 are shifted by the same amount, the sound is called an inharmonic complex and the pitch is shifted linearly from F0. It suggests that the pitch is not determined by the difference between adjacent frequency components. Our model reproduces the linear shift in pitch for such inharmonic complexes (Section 3.2). Further, we test the model's sensitivity to the phase relationship among harmonics, using Schroeder phase, alternating phase and random phase (Section 3.3). By changing the phase relationships of a harmonic complex, we can change the waveform envelope of the complex without changing its spectral content. In general, the pitch of resolved harmonics is F0 and does not depend on the phases while the envelope cue is dominant for unresolved harmonics. Lastly, we test one kind of pitch-evoking stochastic stimulus, iterated ripple noise (IRN), which has peaks in spectra but little waveform modulation (Section 3.4). IRN stimuli can have multiple pitches depending on the number of iterations. More details of the various sound stimuli can be found in reviews Plack and Oxenham (2005) and Schnupp et al. (2011, see Sections 3.2, 3.3).

### 3.1. Detecting the missing fundamental

We first consider estimating the pitch of a missing fundamental (MF) harmonic complex. To understand the essential mechanism, we first use an idealized AN model for illustration and later a more detailed AN model to compare with psychophysical and physiological data. The pitch of a complex of harmonics is its fundamental frequency (F0), regardless of whether F0 is present in the sound or not (Schouten, 1938; Licklider, 1954). The pitch information is present implicitly in the harmonic relationship among frequency components; F0 is the largest common factor of harmonics. Since the resolved harmonics excite distinct places on the basilar membrane (Plack and Oxenham, 2005, see Figures 2.3), each AN fiber is modulated only by the frequency component of the sound that is close to its best frequency (BF). For example, in response to an MF harmonic complex of frequencies {3F0, 4F0, 5F0}, AN fibers of BFs 3F0, 4F0 and 5F0 are stimulated primarily. The mean excitatory current from AN fibers of BF can be approximated as a half-wave rectified sinusoidal wave of the same frequency (Figure 1), which is referred to as an idealized AN model. The half-wave rectification approximates hair cell transduction and the idealized AN model assumes sharp frequency filters. Therefore, the pitch (F0) is not explicit in the firing patterns of the AN fibers. Information from different frequency channels of AN needs to be combined to compute F0.

In our model, an SD unit in the second stage receives a weighted sum of AN inputs from a broad range of frequency channels (Figure 1, details see Methods). Since the common divisor of the harmonic frequencies is F0, the sum of AN inputs has a periodicity of 1/F0 with a fast-rising, high-peaked component followed by two smaller peaked components in each cycle (Figure 1, total input trace). The SD units can phase-lock to these fast, large transient input components and fire with the same frequency as the pitch of the sound. Hence, the regular firing pattern of SD units can serve as a neural representation of pitch.

We use a biophysical cellular model (Meng et al., 2012) for the SD units, a reduced version of the Type II model in Rothman and Manis (2003) that was originally designed for neurons in the ventral cochlear nucleus. An SD unit only fires once when input rises fast enough and does not fire repetitively for slow or steady input (Figure 2A). This phasic firing property is due primarily to a low threshold potassium current (*I*_*KLT*_) that activates with a time constant that is comparable to that of the resting membrane potential. If a neuron depolarizes slowly, *I*_*KLT*_ will be activated and effectively increase the threshold of firing. If the membrane potential changes fast, on the other hand, and crosses the threshold before *I*_*KLT*_ has time to activate, an action potential is generated. After an action potential, *I*_*KLT*_ is strongly activated and prevents further spiking. Due to its phasic property, an SD unit has a band-pass frequency tuning for half-wave rectified-sinusoidal inputs (Meng et al., 2012, see Figure 5A). The amplitude threshold for low frequency sinusoidal inputs is much higher since the rising part within each cycle of low frequency inputs is too slow. On the other hand, an SD unit also does not respond to high frequency inputs due to the lack of resting period (with no input) for the SD unit to recover from the activated *I*_*KLT*_. Further, an SD unit fires only one spike per cycle with high vector strength (Equation 8), except for inputs at high frequencies when the SD unit starts skipping cycles and its firing rate declines steeply with frequency.

To compare with psychophysical results of pitch perception, we use a detailed AN model developed by Meddis et al. (2013) to generates spike trains of AN fibers from multiple BF channels simultaneously (details see Methods). Temporal patterns of the AN spike trains reflect individual harmonics within the MF complex. Since lower-order harmonics are well separated in the cochlear, the AN fibers are modulated mainly by the harmonic frequency close to their BF, similar to the simple approximation of AN activities as rectified sine-waves (Figure 1). In response to an MF complex of frequencies {600, 800, 1000} Hz (F0 = 200 Hz), for example, the AN fibers of BFs lower than 600 Hz have repetition rates at about 600 Hz, those of BF = 800 Hz have repetition rate at 800 Hz and those of BFs higher than 1000 Hz have repetition rate at about 1000 Hz (Figure 2C). There is little interaction between harmonics in the activities of AN fibers from the same BF channel for this stimulus in the AN model that we use (Lopez-Poveda and Meddis, 2001).

The SD units can phase-lock at F0 by detecting the coincidence among the AN spike trains across frequency channels. The total synaptic input to one of the SD units at CF = 200 Hz, from the AN fibers of BF at 200 Hz and above (Equation 7), has a large peak followed by two smaller peaks in each cycle of 1/F0 (Figure 2D), similar to the sum of rectified sine-waves (Figure 1, total input trace). Note that the CF of an SD unit refers to the frequency of AN fibers from which it receives the largest input; this CF should not be confused with the characteristic frequency as commonly used. The majority of the input to this SD unit is modulated at 600 Hz (3F0), reflected as three peaks in the averaged inputs within a cycle of 1/F0 (Figure 2F, blue), from BF channels lower than 600 Hz. This is due to the larger weights (Figure 2C, red bars) on AN inputs of low BFs that converge on this SD unit. The component of 800 Hz, from BF channels 600 to 1000 Hz, coincides with the first peak of the 600 Hz component in each cycle (Figure 2F, green), thus making the rising part of the first peak steeper. The phase of the 1000 Hz component, from BF channels higher than 1000 Hz, is slightly in advance to the other two components (Figure 2F, red). The components of 800 and 1000 Hz are out of phase with the following two peaks of the 600 Hz component input, making their rising parts shallower and rendering it more difficult for the SD unit to respond to the following two peaks of input in each cycle. As a result, the SD unit fires precisely and reliably almost once every cycle of 1/F0 to the MF complex (Figure 2E).

Consequently, the summed ISI histogram over CF has a large peak at 1/F0, corresponding to a decoded pitch of F0 for the MF complex (Figure 2G). In particular, the SD units across CF sites generally show a large peak at 1/F0 (Figure 2G), which means that pitch is represented by SD units across a broad range of CFs. In contrast to most autocorrelation (AC) models (Meddis and Hewitt, 1991a,b) which sum over all AC functions of AN spike trains across BF to decode pitch, each SD unit combines inputs from AN fibers across BF and extracts the shared periodicity among those AN fibers. Note the reduced peak around CF of 500 Hz in Figure 2G. It results from comparable amplitudes of the 1000 Hz component of the input with those of the 600 and 800 Hz components to SD units. The difference in phase results in a shallower rising part of the first peak of input in each cycle, resulting in some skipped cycles.

#### 3.1.1. The pitch strength decreases with the lowest harmonic number

**Figure 3:**
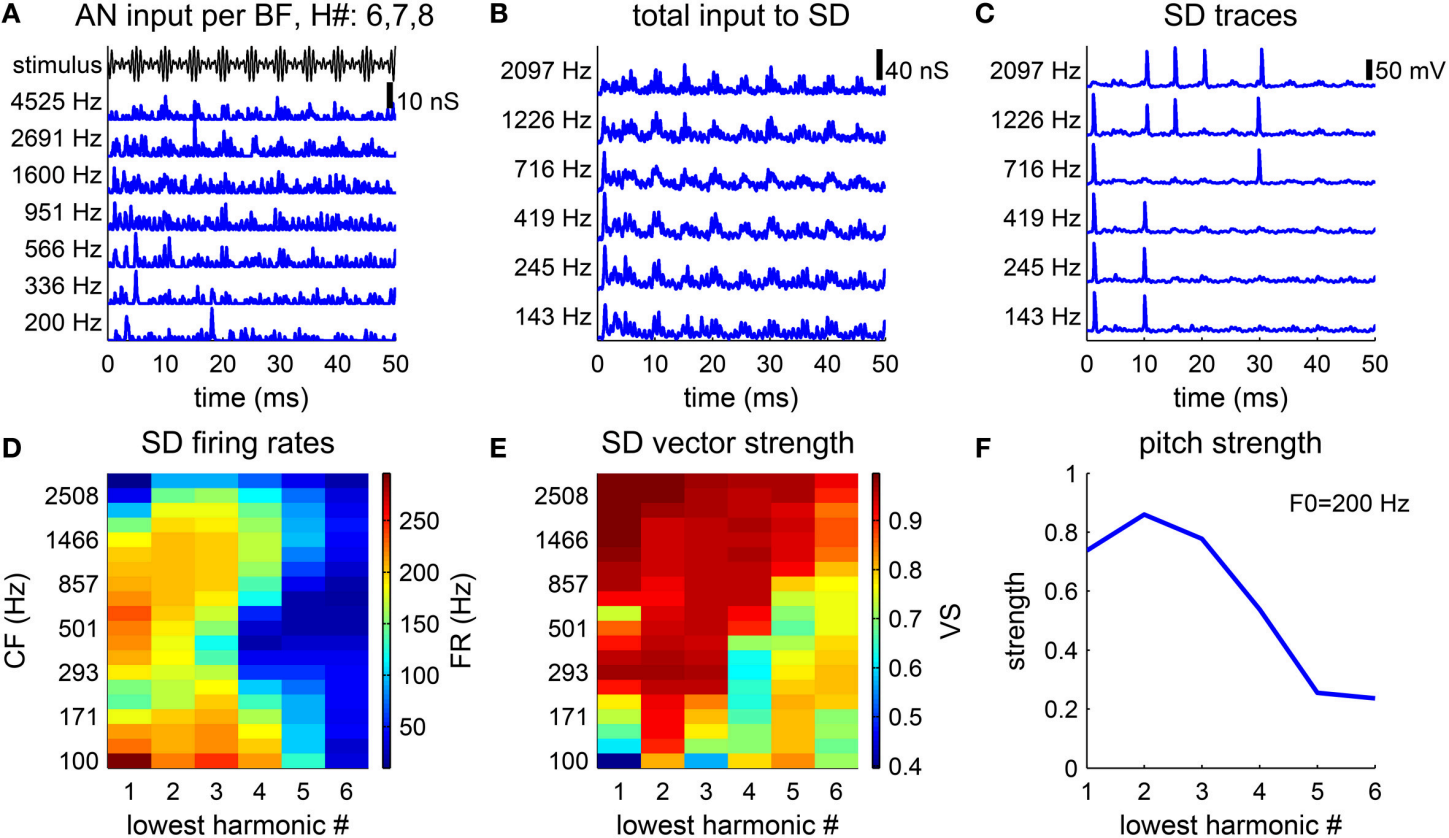
**Pitch strength is lower for higher-order harmonic complexes**. **(A–C)** Model response to a harmonic complex of harmonic number {6, 7, 8} and fundamental frequency F0 = 200 Hz. **(A)** AN conductance inputs (blue), *g*_*AN*_ (Equation 5), from representative BF channels are modulated by the stimulus envelope (black). **(B)** Total conductance inputs, *g*_*syn*_ (Equation 4), AN inputs as shown in **(A)** weighted by the footprint ω (Equation 7), to SD units at different CFs (left). **(C)** SD units phase-lock to the stimulus, but fail to fire at every cycle. **(D,E)** Responses to harmonic complexes with 3 consecutive harmonics of F0 = 200 Hz. The lowest harmonic number varies from 1 to 6. **(D)** Firing rates of SD units across CF decrease for higher-order harmonics. **(E)** Same as D for vector strength (Equation 8). **(F)** Pitch strength decreases as the lowest harmonic number increases.

The pitch perception of an MF complex depends on the harmonic number indices. Resolved harmonics generally give stronger pitch sensation. Pitch salience is weaker if the MF complex contains only higher-order harmonics (Moore and Moore, 1982). The waveform of an MF complex of harmonic number {6, 7, 8} (Figure 3A, black) is modulated more gradually than an MF complex of harmonic number {3, 4, 5} (Figure 2B) with the same F0. In response to the MF complex of harmonic number {6, 7, 8}, the AN inputs, *g*_*AN*_ (Equation 5), from high BFs are modulated by the temporal envelope of the MF complex. The spike times are distributed broadly in phase within each cycle of 1/F0 (Figure 3A, blue). The fine structures of the AN inputs are of high frequency, which reflect the frequency components within the MF complex. The total conductance input, *g*_*syn*_ (Equation 5), to each SD is slowly-modulated at the F0 (Figure 3B). The SD units fail to fire at many cycles (Figure 3C), since their AN inputs do not rise fast enough in those cycles. Moreover, the spreading of the AN inputs keeps *I*_*KLT*_ partially activated, which effectively raises the threshold of firing for the SD units.

The decoded pitch strength decreases with the lowest harmonic number (Figure 3F), which qualitatively agrees with the decrease in pitch salience in listeners' reports (Moore and Moore, 1982). There are two factors contributing to the decrease. First, the firing rates of the SD units across CF are lower when the lowest harmonic number is higher (Figure 3D). A lower firing rate results in more ISIs that are longer than the period of 1/F0. The pitch-selective neurons from auditory cortex measured by Bendor and Wang (2005) also show a decrease in firing rates to higher-order harmonic complexes. Second, the vector strength (Equation 8) (with respect to the period of 1/F0) of the SD units at low CF deteriorates for higher-order harmonics (Figure 3E). The SD units at high CF can maintain high vector strength, since the AN fibers of high BF are better modulated by the envelope of the stimulus.

#### 3.1.2. Tuning curves for pure tones and the MF complexes

**Figure 4:**
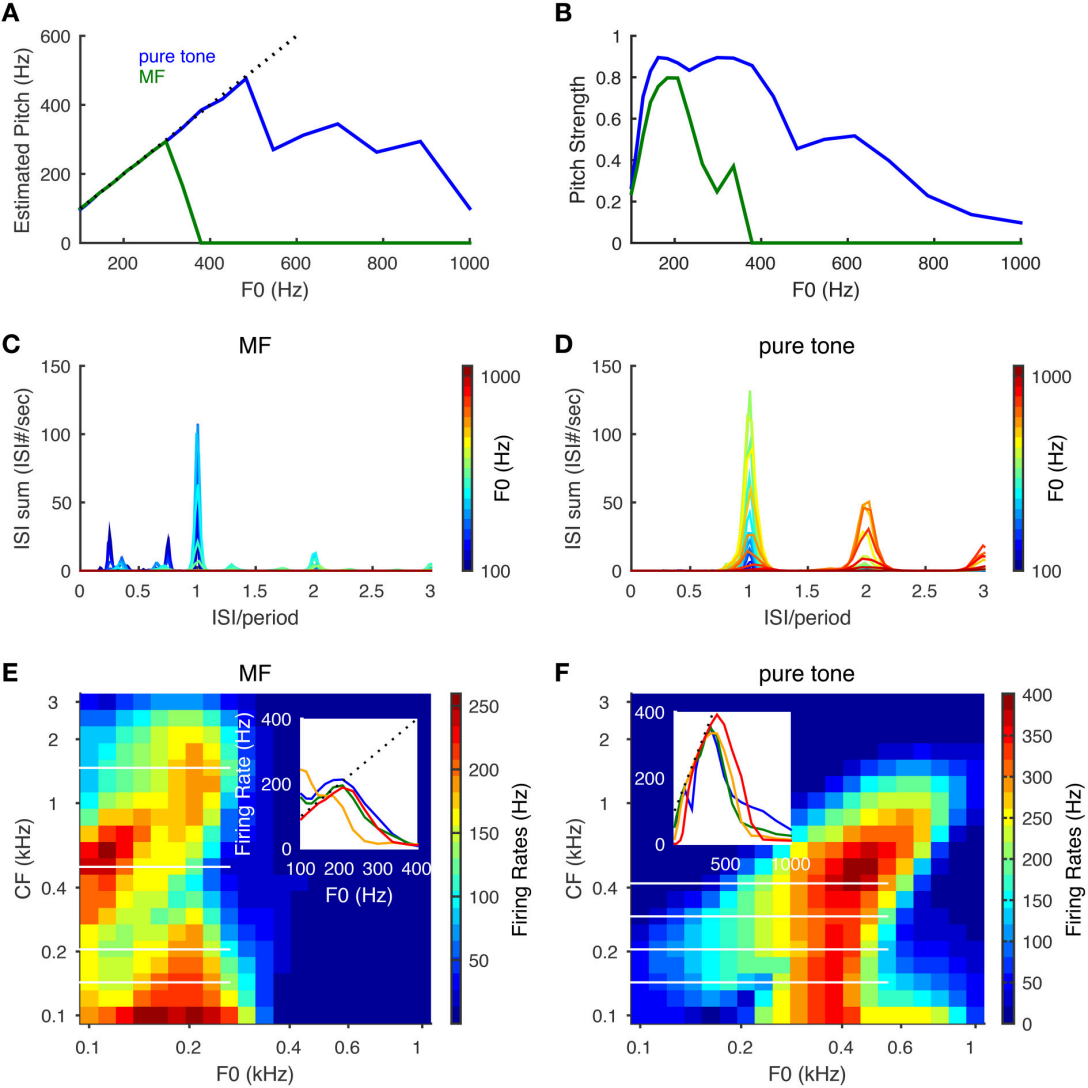
**Pitch frequency estimation and firing rate tuning of SD units for pure tones and MF complexes**. Harmonic numbers for MF complexes are {3, 4, 5} with fundamental frequency, F0, varying logarithmically from 100 to 1000 Hz. For pure tones, F0 refers to tone frequency. **(A)** Estimated pitch is the same as tone frequency for pure tones (blue) up to about 500 Hz and F0 for MF complexes (green) for F0 up to 300 Hz. Dotted black line indicates equality with F0. **(B)** Same as **(A)** for pitch strength. **(C)** The summed ISI histograms of SD units over CF in response to MF complexes of different F0 (color coded). The *x*−axis is ISI normalized by the period (1/F0) of the MF complexes. **(D)** Same as **(C)** for pure tones. **(E)** Firing rates of SD units across CF in response to MF complexes as a function of F0. The *x*− and *y*− axes are on a logrithmic scale. Inset: representative firing rate tuning curves as a function of F0 for SD units of CFs 143 Hz (blue), 205 Hz (green), 501 Hz (orange), 1466 Hz (red). The positions of these four CFs are shown as white horizontal lines in the heat plot. **(F)** Same as **(E)** for pure tones. Inset: representative firing rate tuning curves as a function of F0 for SD units of CFs 143 Hz (blue), 205 Hz (green), 293 Hz (orange), 419 Hz (red). Results are averaged over 50 runs with independent AN inputs. Pitch and pitch strength are set to zero when the mean firing rate of all SD units is below 15 Hz.

The model can estimate pitch frequency accurately for both pure tones and the MF complexes of low pitch frequencies. The estimated pitch of a pure tone is identical to the tone frequency up to about 500 Hz (Figure 4A, blue) with high pitch strength (Figure 4B, blue) and that of the MF complexes is the same as the missing fundamental (F0) up to about 300 Hz (Figures 4A,B, green). The summed ISI histograms of the SD units across CF show a dominant peak at the period (1/F0) for tones of low F0s. As F0 increases, the SD units are more likely to skip cycles, resulting in more ISIs at multiples of the period (Figures 4C,D). For an MF complex of low F0 (around 100 Hz), some SD units can respond to the two smaller peaks in the summed AN input that they receive in each cycle of F0 (Figure 2F) and fire more than once in some cycles, resulting in ISIs that are less than a period (Figure 4C, dark blue). As F0 increases, it becomes harder for the SD units to fire more than once within a cycle, hence the magnitudes of the two small peaks at ISIs less than a period are reduced.

**Figure 5:**
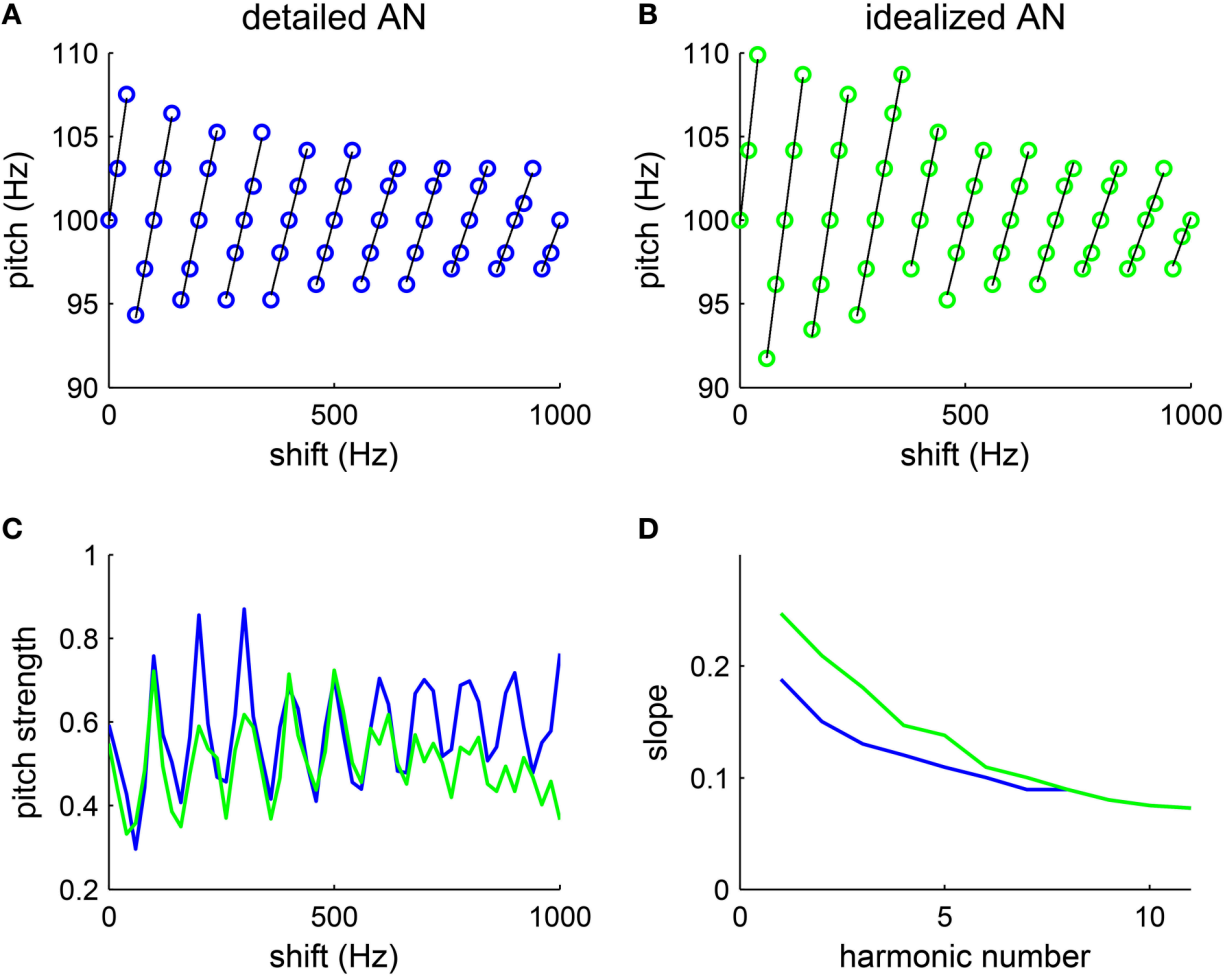
**Pitch varies with the amount of frequency shift of inharmonic complexes**. The tones are generated by shifting all harmonic components (F0 = 100 Hz, harmonic # 1–6) by the same amount Δ*f* (see Methods). **(A,B)** The decoded pitch shifts linearly with the frequency difference from the closest harmonic (fitted lines are in black), consistent with psychophysical results (Patterson and Wightman, 1976, see Figures 1, 2). The decoded pitch is discontinuous at half-way between harmonics, suggesting ambiguous pitch. **(A)** With a detailed AN model, we use the matlab package (MAP1-14h) developed by Meddis et al. (2013) to generate AN spike trains (details see Methods). For each Δ*f*, one trial was run with stimulus duration 100 ms. **(B)** With an idealized AN model, AN inputs were approximated by rectified sine-waves. Specifically, the AN input current to an SD unit of CF *f*_*SD*_ is $I\left(t\right)=A\overset{6}{\underset{n=1}{\sum }}\omega \left(nF0+\Delta f,f_{SD}\right){\left[\sin\left(2\pi \left(nF0+\Delta f\right)t\right)\right]}^{+}$, where [·]^+^ = max(·, 0). The footprint function ω is the same as that in the detailed model (Equation 7) and the amplitude is *A* = 500 pA. White noise with intensity 100 nA was added to the input current in simulations. For each Δ*f*, one trial was run with stimulus duration of 200 ms. The increment in Δ*f* was 20 Hz in both **(A,B)**. **(C)** The pitch strength is strongest when the frequency shift Δ*f* is a multiple of F0, in which case the complex becomes harmonic, and pitch strength weakest when Δ*f* is half-way between harmonics. Both the detailed AN model (blue) and the idealized AN model (green) give similar results. **(D)** The slopes of the fitted lines (black) in **(A)** (blue) and **(B)** (green) decrease monotonically with the lowest harmonic number in a complex.

The firing rates of the SD units show band-pass and low-pass tuning for the MF complexes and only band-pass for pure tones. For the MF complexes, the SD units at intermediate CF (about 300–800 Hz) show low-pass tuning for F0 (Figure 4E inset, orange curve), while the SD units at lower and higher CFs show band-pass tuning (Figure 4E). For pure tones, most SD units across CF show band-pass tuning of firing rates for tone frequencies (Figure 4F). The firing rate of a band-pass SD unit increases with F0 near linearly until it reaches maximum (Figures 4E,F, insets), since it is entrained to fire at the pitch frequency F0. Band-pass SD units peak around F0 = 200 Hz for MF complexes and mostly around 400 Hz for pure tones. The firing rate drops at higher F0 since the SD unit needs time to recover from *I*_*KLT*_. The frequencies where these peaks occur reflect intrinsic resonant properties of such SD units (Meng et al., 2012, see Figure 5A) (Remme et al., 2014) and these frequencies depend on the temporal profile of the input. The band-pass peak's location for higher-CF SD units shifts toward higher tone frequency for pure tones (Figure 4F), since the frequency region of activated AN neurons increases with tone frequency. The SD units entrain better (i.e., over a large F0 range) to pure tones than MF complexes since the total AN input in response to a pure tone has only one peak within a cycle rather than multiple peaks as the case for the MF complexes (Figure 2F). Note that the SD units can respond to MF complexes with frequency components outside their response tuning curves of pure tones. In other words, these SD units respond to the pitch rather than the frequency components of some MF complexes. For example, an SD unit at CF = 205 Hz fires almost maximally to the MF complex of F0 = 200 Hz, but fires little to the individual frequency components, {600, 800, 1000} Hz, when presented alone as pure tones (Figures 4E,F). Hence our SD units satisfy the criteria for pitch-selective neurons used by Bendor and Wang (2005). Those pitch-selective neurons are also mainly found in the low-frequency region with preferred fundamental frequencies around 200 Hz (Bendor and Wang, 2005, see Figure 3b).

### 3.2. Pitch shift of equally spaced inharmonics

An equally-spaced inharmonic complex with spacing F0 consists of harmonics of F0 shifted by Δ*f* (see Methods); it evokes a pitch that is shifted away from F0. Interestingly, the shift in pitch from F0 is linearly related with Δ*f* (Schouten, 1940; de Boer, 1956; Patterson, 1973; Patterson and Wightman, 1976). Specifically, when the first component of the inharmonic complex was above the closest harmonic frequency, i.e., mod (Δ*f, F*0) < *F*0/2, the matched pitch was higher than F0. Conversely, when the first component of the inharmonic complex was below the closest harmonic frequency, i.e., mod (Δ*f, F*0)>*F*0/2, the decoded pitch was lower than F0. When Δ*f* was a multiple of F0, the complex became harmonic and the pitch was the fundamental frequency F0. When the inharmonic complex was shifted about halfway between harmonics, i.e., mod (Δ*f, F*0)≈*F*0/2, listeners perceived an ambiguous pitch, which was reflected in a bimodal or multi-model distribution of pitch matches. Moreover, the slope of the pitch dependence on *Deltaf* decreases as the lowest frequency increases. (Patterson, 1973; Patterson and Wightman, 1976).

Our model reproduces similar pitch dependence on the frequency shift Δ*f* as that observed in their psychophysical experiments. The shift in pitch from F0 varies linearly with the frequency shift from the closest harmonic (Figure 5A, black lines are fitted lines). The slope of the linear dependence of pitch on Δ*f* (Figure 5A, black) decreases as the lowest harmonic number increases (Figure 5D, blue). The result is quantitatively consistent with that measured in the psychophysical experiments (Patterson and Wightman, 1976, see Figure 3).

The decoded pitch strength also varies with frequency shift Δ*f*. Pitch is the strongest when Δ*f* is a multiple of F0, in which case the complex is harmonic (Figure 5C). The inharmonic complexes shifted half-way between harmonics give the lowest pitch strength. Those Δ*f*s with lowest pitch strengths are where pitch perception changes discontinuously, consistent with the ambiguity in pitch perception in listeners' reports (Patterson, 1973). Hence, the model can qualitatively account for the trend in pitch strength reported.

With an idealized AN model, where the average synaptic input currents from the AN fibers are approximated with rectified sine-waves, the SD units can also estimate the pitch of the inharmonics with a similar dependence on frequency shift (Figure 5B). A frequency shift in an inharmonic complex results in a slight temporal difference in the rising phase within each cycle of input. By phase-locking to the rising part of the input with high temporal precision, the SD units can encode the small pitch difference for different frequency shifts. Pitch strength computed with the idealized model (Figure 5C, green) also varies linearly with the frequency shift Δ*f* as that computed with the detailed model (Figure 5C, blue). Moreover, slopes of the fitted lines (Figure 5B, black) also decrease with the lowest harmonic number (Figure 5D, green), though the slopes at low harmonic numbers are slightly higher than those computed with the detailed AN model. Note that there is no frequency bandwidth in the idealized model, which means that all frequencies are resolved. Hence the decrease in slopes computed with the idealized model is not due to the degradation in resolvability of higher order harmonics. This suggests that the temporal interaction of equally spaced frequencies can account, to a large degree, for the observed pitch shift pattern, with minimum nonlinearity in AN processing.

### 3.3. Phase sensitivity

In this section, we test the model's performance on different phase relationships of harmonic complexes. Earlier temporal models that use waveform fine structures (de Boer, 1956; Schouten et al., 1962) were criticized for producing greater phase sensitivity than was observed (Wightman, 1973b). Listeners are insensitive for most phase variations in resolved harmonics, however, relative phase in unresolved harmonics can affect pitch perception (Mathes and Miller, 1947; Houtsma and Smurzynski, 1990; Shackleton and Carlyon, 1994) The spectral models are phase-insensitive since they only make use of the spectral content of sound. AC models are insensitive to phase variations within a frequency channel but depend on phase relationships among channels and these models can predict some phase-sensitivity as observed in experiments (Meddis and Hewitt, 1991b; Meddis and O'Mard, 1997). We test our model in the following three phase relationships: Schroeder phase, alternating phase and random phase. A Schroeder phase relationship reduces the envelope modulation, thus reducing temporal cues of the stimulus; an alternating phase stimulus makes the repetition rate of the envelope different from the fundamental frequency; random phases in resolved harmonics have little effect on pitch perception.

#### 3.3.1. Schroeder phase

**Figure 6:**
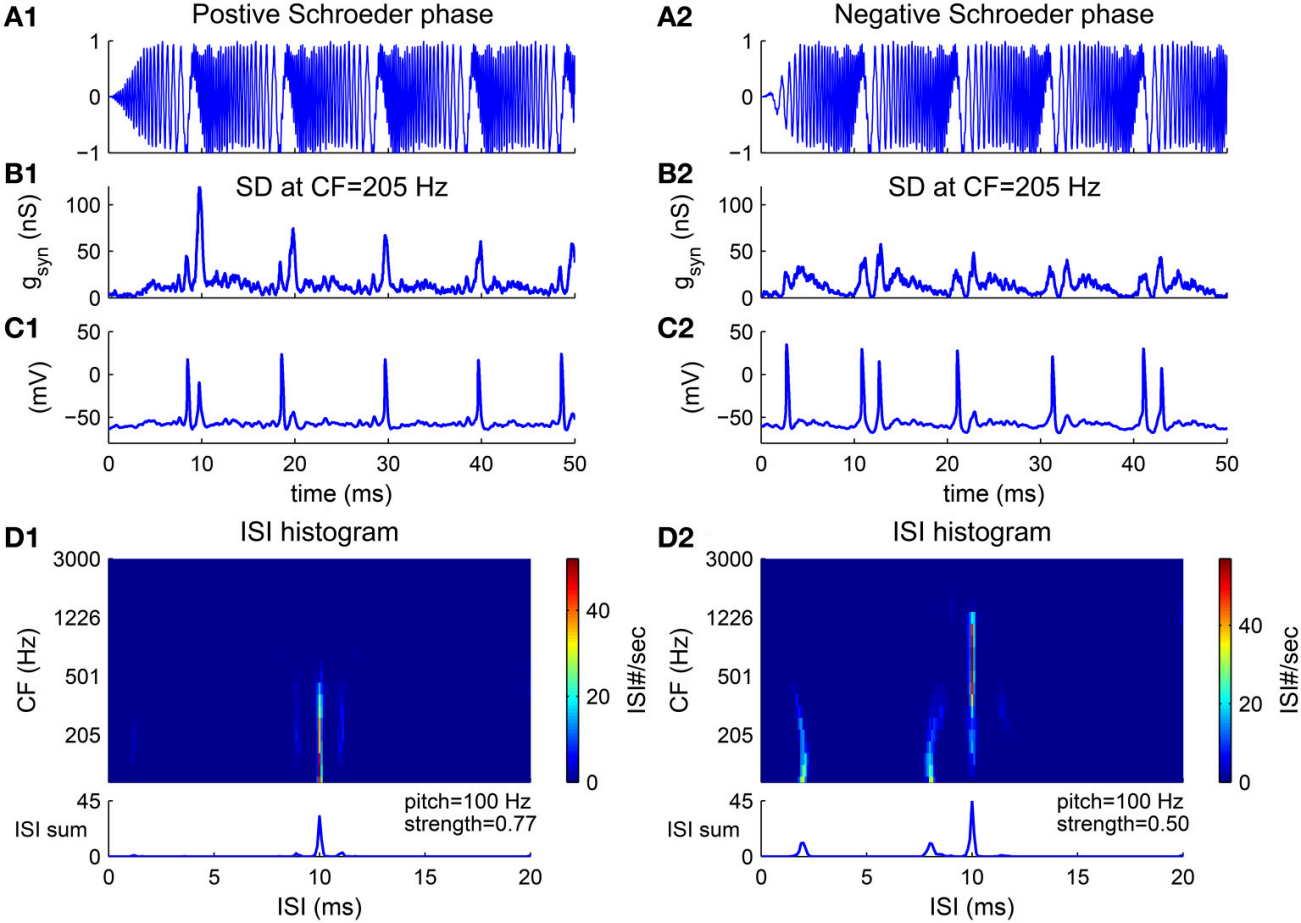
**Pitch frequency estimates of harmonic complexes with positive Schroeder phase (***m***_**+**_, A1–D1) and negative Schroeder phase (***m***_**−**_, A2–D2) (F0 = 100 Hz)**. **(A)** Stimulus waveforms (normalized). Schroeder phase relationships result in relatively flat envelopes. An *m*_+_ complex has decreasing instantaneous frequency within each period while an *m*_−_ complex is a time reversal of *m*_+_ with increasing instantaneous frequency within each period (details see Methods). **(B)** Total synaptic input *g*_*syn*_ generated from convergent AN fibers to a representative SD unit at CF = 205 Hz (Equation 4) is modulated at F0. *g*_*syn*_ is peakier to an *m*_+_ than to an *m*_−_ complex (Kohlrausch and Sander, 1995). **(C)** The SD unit phase-locks at F0, in response to input shown in **(B)**. **(D)** First order ISI histograms of SD units across CF. Sums of ISI histograms over CF are shown at bottom. Decoded pitch for both *m*_+_ and *m*_−_ complexes are their fundamental frequency F0 = 100 Hz. Two small peaks at shorter ISIs for *m*_−_ **(D2)** are due to double spikes within a cycle, as can be seen in **(C2)**. This results in a weaker pitch strength for an *m*_−_ complex than for an *m*_+_ complex.

An harmonic complex with Schroeder phase has a relatively flat temporal envelope using a constant curvature (second derivative) in phase with respect to frequency (Equation 10) (Schroeder, 1970). Schroeder phase complexes have been used to investigate how important the cue of waveform envelope is in pitch perception. F0 discrimination of Schroeder phase complexes is similar to that of harmonic complexes with sine phase for resolved harmonics but poorer for unresolved harmonics (Houtsma and Smurzynski, 1990), suggesting that the temporal envelope is not a determining cue for resolved harmonics. A positive Schroeder phase complex (*m*_+_) (Figure 6A1) has a monotonically decreasing instantaneous frequency within each period. A negative Schroeder phase complex (*m*_−_) (Figure 6A2) is similar to an *m*_+_ complex but reversed in time. Although an *m*_+_ complex has the same long-term spectral power and similar temporal envelope as an *m*_−_ complex, it produces less masking in tone detection tasks (Kohlrausch and Sander, 1995; Lentz and Leek, 2001).

The model estimates the pitch of both *m*_+_ and *m*_−_ complexes as their fundamental frequency. The total AN input to a representative SD unit is modulated at F0 for the Schroeder phase complexes despite their flat temporal envelope (Figures 6B1,B2). One reason is that the AN input to the SD unit only contains information of a small number of harmonics due to the limited footprint width (σ = 2 octaves in Equation 7). Another reason is that the phase dispersion of the basilar membrane can change the phase relations in AN fibers, resulting in a higher modulation amplitude. It has been shown that the basilar membrane has a negative phase curvature which would give a peakier output to an *m*_+_ than to an *m*_−_ complex (Kohlrausch and Sander, 1995; Rhode and Recio, 2000). In our model, the AN input to the SD unit is also peakier for the *m*_+_ than for the *m*_−_ complex (Figure 6B). Therefore, the SD unit fires more precisely in each cycle to the *m*_+_ complex (Figure 6C1), but has double spikes in some cycles to the *m*_−_ complex (Figure 6C2). The double spikes of the SD units at low CF in response to the *m*_−_ complex result in two small peaks at ISIs that are shorter than 1/F0 in the ISI histograms (Figure 6D2). Overall, the model decodes F0 as the pitch for Schroeder phase complexes with a larger pitch strength for the *m*_+_ complex than for the *m*_−_ complex (Figure 6D, bottom).

#### 3.3.2. Alternating phase harmonics

**Figure 7:**
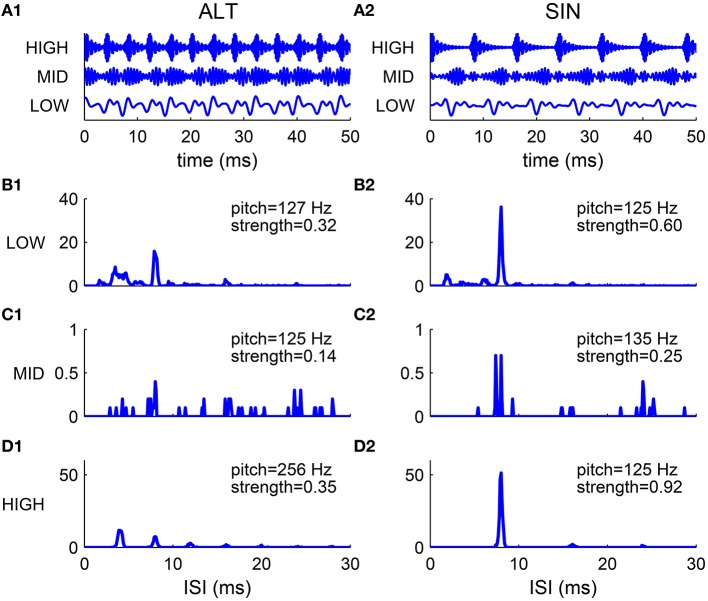
**Comparison of harmonic complexes with an alternating phase (ALT) (A1–D1) and those with a sine phase (SIN) (A2–D2) in different frequency regions**. **(A)** Normalized stimulus waveforms of ALT **(A1)** and SIN **(A2)** complexes (F0 = 125 Hz) filtered in LOW (125–625 Hz), MID (1375–1875 Hz) and HIGH (3900–5400 Hz) frequency regions (extended frequency ranges for AN and SD units are used, for details see Methods). Envelope repetition rate of an ALT complex is one octave higher than its fundamental frequency, while envelope repetition rate of a SIN complex is the same as its F0. **(B–D)** Sums of ISI histograms of SDs over CF for complexes filtered in LOW **(B1,B2)**, MID **(C1,C2)** and HIGH **(D1,D2)** frequency regions. Both ALT and SIN complexes have a pitch at F0 in the LOW frequency region, while the ALT complex has a pitch one octave higher than the SIN complex in the HIGH frequency region. Complexes in the MID frequency range have low pitch strength and low firing rates. Our modeling results are consistent with the psychophysical results in Shackleton and Carlyon (1994).

The spectral cue of pitch (the F0) is dominant for resolved harmonics, while the envelope cue of pitch is often dominant for unresolved harmonics. This was demonstrated in pitch matching experiments by Shackleton and Carlyon (1994) using harmonic complexes with alternating phase (ALT), where odd harmonics are in sine phase and even harmonics are in cosine phase. The envelope repetition rate of an ALT harmonic complex is twice that of its F0 (Figure 7A1). The stimuli were generated by filtering a complex of 80 harmonics (F0 = 125 Hz) in one of the three spectral regions: (1) LOW, 125–625 Hz; (2) MID, 1375–1875 Hz; (3) HIGH, 3900–5400 Hz. The matching stimuli were harmonic complexes in sine phase (SIN) filtered in the same spectral region as the test stimuli. They found that in the LOW region the ALT complex was matched by the SIN complex of fundamental frequency F0, while in the HIGH region the ALT complex was matched to the SIN complex of fundamental 2F0.

We tested our model using the same stimuli as that used in Shackleton and Carlyon (1994) (see Methods). The decoded pitch of the SIN complex is F0 in all spectral regions (Figures 7B2–D2). The ALT complex in the LOW region has a pitch at F0 while that in the HIGH region has a pitch at 2F0 (Figures 7B1,D1). Both the ALT and the SIN complexes in the MID region have very low firing rate and weak pitch strengths (Figures 7C1,C2), consistent with the dispersed pitch matching histogram in Shackleton and Carlyon (1994, see Figure 2E).

#### 3.3.3. Random phase

Phase changes in resolved harmonics have little effect on pitch perception (Patterson, 1973; Wightman, 1973b; Lundeen and Small, 1984). Since resolved harmonics are separated in the cochlea, there is little interaction between frequency components. Hence, the pitch of resolved harmonics only depends on the spectral content and is insensitive to phases. Since phase changes affect temporal fine structure as well as temporal envelope, as shown in the Schroeder phase and the alternating phase examples above, temporal models that rely on temporal fine structure will be sensitive to phase changes in resolved harmonics (de Boer, 1956; Schouten et al., 1962).

**Figure 8:**
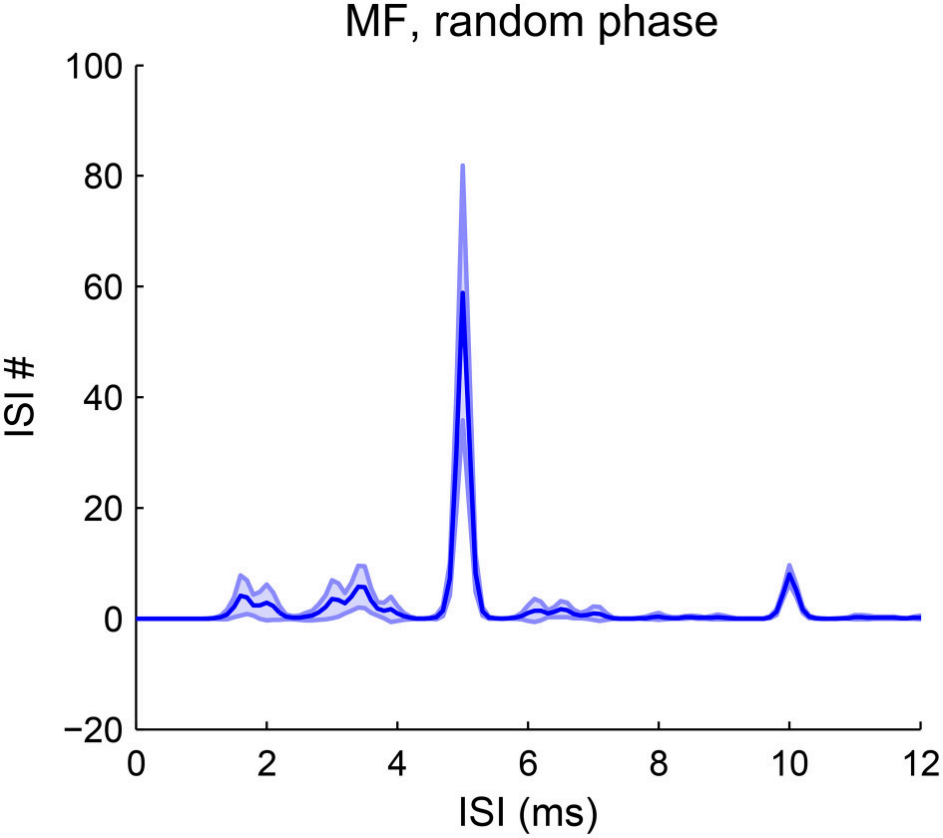
**Pitch frequency estimates of MF complexes with random phase**. The first-order ISI histogram pooled over all SD units across CF shows a major peak at 1/F0 (F0 = 200 Hz). The harmonic frequencies of the MF complexes for all trials are {600, 800, 1000} Hz. Phases for the three harmonics are randomly sampled from [−π, π] independently. The decoded pitch is the same as that for an MF complex in cosine phase with lower strength (Figure 2G). The decoded pitch for all trials is 200 Hz. Pitch strength has a mean 0.54 and standard deviation 0.13. A total of 50 trials were run and the shaded area is within one standard deviation.

Our model is robust to phase variations in resolved harmonics. The estimated pitch is always the fundamental frequency for MF complexes in random phase (Figure 8). The pooled ISI histograms have a predominant peak at 1/F0 in all trials of phase variations. Although phase can vary among AN channels due to both the random phase relationship in the stimuli and the phase dispersion effect of the basilar membrane, the convergence of AN input across channels still results in coincidence at a period of 1/F0. Therefore the model is robust to most phase variations. The pitch strength does vary with the phases, with a mean strength slightly lower than that with cosine phase (Figure 2G). For human listeners, a random-phase harmonic complex also has a slightly weaker pitch strength than a cosine-phase complex (Lundeen and Small, 1984; Shofner and Selas, 2002).

### 3.4. Iterated ripple noise

**Figure 9:**
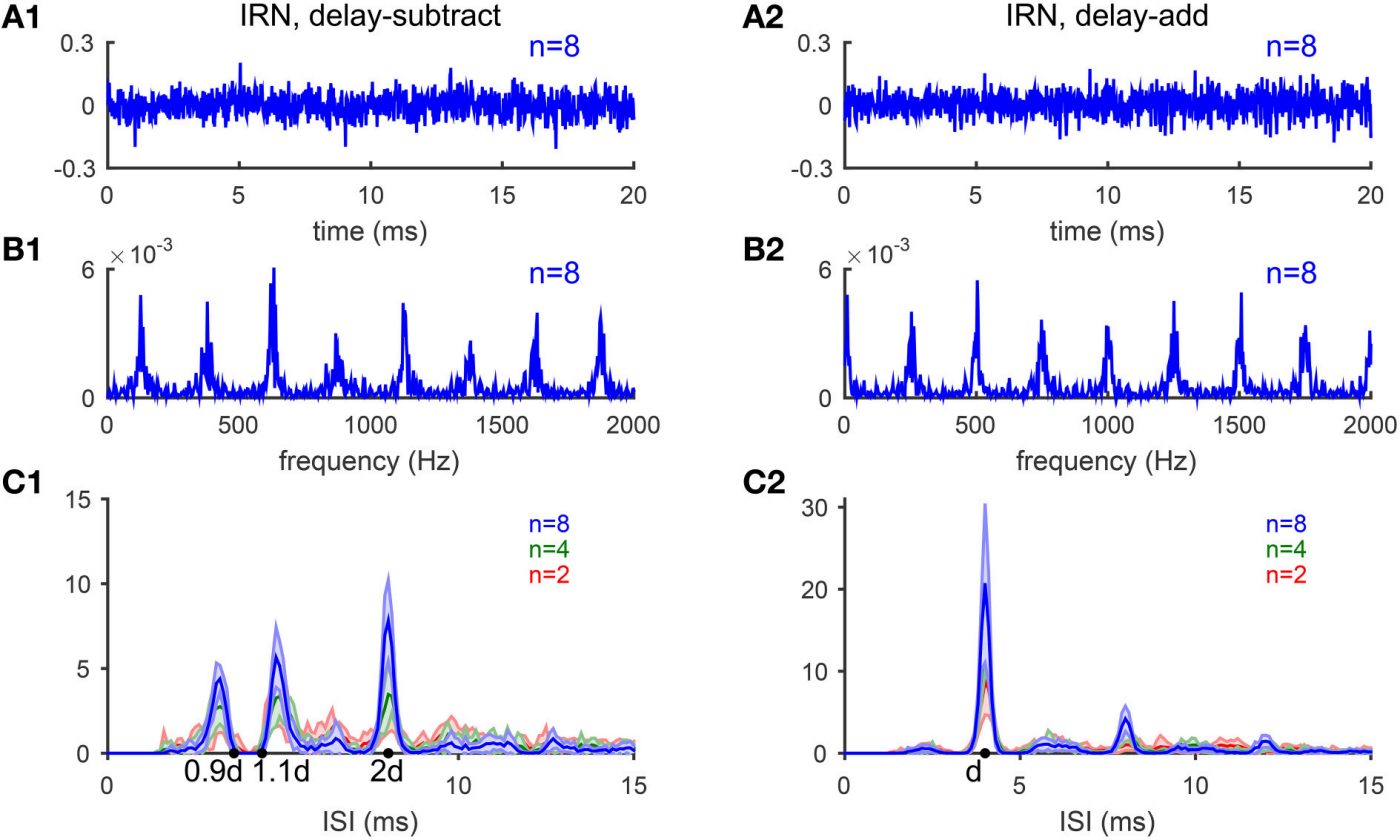
**Iterated-ripple-noise (IRN) stimuli generated with delay-subtract (A1–C1) and delay-add (A2–C2) operations**. **(A1)** Waveform of an IRN stimulus generated by delay-subtract operations (gain *g* = −1, delay *d* = 4 ms and iteration number *n* = 8). **(A2)** Waveform of an IRN stimulus generated by delay-add operations (*g* = 1, *d* = 4 ms, and *n* = 8). **(B1,B2)** Spectra of the IRN stimuli shown in **(A1,A2)**, respectively. **(C1)** IRN stimuli generated with delay-subtract have three peaks near 0.9*d*, 1.1*d*, and 2*d* in the ISI histograms summed over CF (*d* = 4 ms). The three peaks are of comparable heights for *n* = 2 (blue) and *n* = 4 (green), while the peak at 2*d* is higher for *n* = 8 (red). Each ISI histogram is an average of 10 realizations of IRN stimuli (shaded area is within one standard deviation). **(C2)** Same as C1 for IRN generated with delay-add. Only one peak at *d* is dominant in the ISI histograms with amplitude increasing with iteration number *n* (Bilsen and Wieman, 1980; Raatgever and Bilsen, 1992; Yost, 1996).

In this section, we test the model's performance for pitch-evoking stochastic stimuli, iterated ripple noise (IRN), that are generated by a cascade of delay and add (gain *g* = 1) or delay and subtract (*g* = −1) operations on a segment of white noise (see Methods) (Yost et al., 1998). Waveforms of IRN stimuli generated by delay-add and that generated by delay-subtract are indistinguishable and do not have pronounced envelope modulation (Figures 9A1,A2), however, their spectra have peaks at different locations and evoke different pitches. IRN with *g* = 1 and delay *d* ms has peaks at harmonics of 1/*d* kHz (Figure 9B2), while IRN with *g* = −1 has spectral peaks half-way between harmonics of 1/*d* kHz, or equivalently odd integer multiples of 1/2*d* kHz (Figure 9B1). IRN stimuli generated by delay-add and that generated by delay-subtract evoke different pitch perception. The pitch of IRN with *g* = 1 is 1/*d* kHz for any iteration number *n*. In contrast, the pitch of IRN with *g* = −1 depends on *n*; pitch is 1/2*d* kHz for *n* > 4, while ambiguous having two values approximately 1/(1.1*d*) and 1/(0.9*d*) kHz for *n* ≤ 4 (Bilsen and Wieman, 1980; Raatgever and Bilsen, 1992; Yost, 1996). Temporal regularity of an IRN stimulus increases with *n*, so does the salience of its pitch sensation (Patterson et al., 1996).

The estimated pitch of IRN stimuli by our model is consistent with the behavioral results (Yost, 1996). The pooled ISI histograms of SD units in response to IRN stimuli with *g* = −1 have three peaks near 0.9*d* and 1.1*d* and 2*d* (Figure 9C1), consistent with the three peaks shown in pitch-matching histograms of human listeners (Yost, 1996). When *n* = 2 or 4, the heights of the three peaks are comparable, suggesting ambiguous pitch perception. When *n* = 8, the peak at 2*d* is higher than the other two peaks, suggesting a dominant pitch at 1/2*d* for large *n* (Figure 9C1). In contrast, the pooled ISI histograms for IRN stimuli with *g* = 1 have only one dominant peak at *d* for all *n*'s, corresponding to a pitch at 1/*d* kHz (Figure 9C2). All ISI histograms show an increase in peak amplitudes with the iteration number *n*, consistent with the increase in pitch salience with *n* (Patterson et al., 1996).

## 4. Discussion

We developed a neuronal network model for pitch frequency representation as a basis for pitch estimation. Our network model is a type of temporal pitch model, but in contrast to previous models (Licklider, 1951; Meddis and Hewitt, 1991a,b) it does not involve the computation of autocorrelation functions that require delay lines. The circuit is feedforward, consisting of a network of biophysically-based slope-detectors (SD) that encode the shared periodicity among the convergent AN inputs. The SD units can phase-lock at a rate that corresponds to the sound's pitch. Hence, the pitch frequency can be estimated from their first-order ISI histograms. The SD units are temporally precise due to their phasic firing properties; they do not fire repetitively within each pitch-related period. Our model can estimate the pitch of the missing fundamental, reproduce the pitch variation with respect to the frequency shift of inharmonic complexes and also account for a large degree the pitch sensitivity/insensitivity to phase relationships in various harmonic complexes. Moreover, the model can also estimate multiple pitches in the case of the iterated-ripple-noise stimuli, with the same dependence on the iteration number as observed in pitch-matching experiments (Yost, 1996).

### 4.1. Physiological correlates

The biophysical model for our slope-detectors was originally developed to account for the phasic discharge pattern of bushy cells of the cochlear nucleus (Rothman and Manis, 2003). The bushy cells show onset response and have better phase locking and entrainment for both on-CF and off-CF pure tones than those of the AN fibers (Joris et al., 1994a,b). Many cochlear nucleus neurons show enhanced representation of pitch-related period in their first-order intervals compared to that of the AN responses (Rhode, 1995). Similar to our SD units, octopus cells receive a broad range of AN input in their long dendrites and show extraordinary temporal precision (Oertel et al., 2000). They show especially strong phase-locking to the F0 of harmonic complexes (Rhode, 1998). Our model shows that neurons with high phase-locking capability are suitable to extract shared periodicity among AN fibers across CF and can encode pitch in their first-order ISIs for a variety of sound stimuli. Other mechanistic models that have phasic or onset properties can also be used as slope-detectors. The particular model (Meng et al., 2012) that we use has demonstrated phasic properties common in auditory brain stem neurons. The time constants of the slope-detectors determine the frequency range of phase-locking. When input rising slopes are shallower, i.e., when synaptic currents are slower (larger τ_*E*_ in Equation 6), a low-CF SD fires less and the pitch is extracted by the SD units from high CF sites where AN inputs are modulated by the sound's envelope (Supplementary Figure 1F). Besides, the footprint from AN to SD (Equation 7) does not need to be one-sided. A symmetric footprint would activate a broader range of SD units. The footprint only needs to be broad enough to combine information of multiple harmonics.

Moreover, our SD units satisfy the pitch-selective criterion used by Bendor and Wang (2005) to characterize pitch-selective neurons in auditory cortex, in that they respond to the MF complexes of frequencies outside their receptive fields. Since the amplitude threshold for an SD unit to phase-lock for higher frequency input increases dramatically (Meng et al., 2012), the SD unit does not respond to the individual frequency components of a harmonic complex when presented alone (Figures 4E,F). When multiple harmonics are presented together, the common divisor, F0, is within the phase-locking range of the SD units. Hence, the SD units can phase lock at the F0 although not responsive to the individual harmonics. As most neurons in the auditory cortex, those pitch-selective neurons fire asynchronously, which is a major difference from our SD units. Our SD units are, therefore, likely to be upstream neurons to those pitch-selective neurons in auditory cortex.

### 4.2. Comparison with autocorrelation models

Many temporal pitch models involve computing the autocorrelation (AC) functions of AN spike trains (Licklider, 1951; Meddis and Hewitt, 1991a,b; Slaney and Lyon, 1993; de Cheveigné, 1998; Balaguer-Ballester et al., 2008, 2009). The AC function of each AN fiber often reflects both the frequency components near its CF and the F0 of the sound (Cariani, 1999, see Figure 4), suggesting that the pitch-related periodicity is shared among AN fibers across CF. The peak at the inverse of the pitch frequency emerges when the AC functions from all AN frequency channels are pooled together (Meddis and Hewitt, 1991a,b; Cariani and Delgutte, 1996a,b). Our SD units extract the shared periodicity among AN frequency channels. The pitch representation in the first-order ISI histogram is, hence, greatly enhanced compared to that of AN responses. Since most of the SD units that are activated by the stimulus phase-lock at F0, pitch can, in principle, be decoded from only a few SD units.

**Figure 10:**
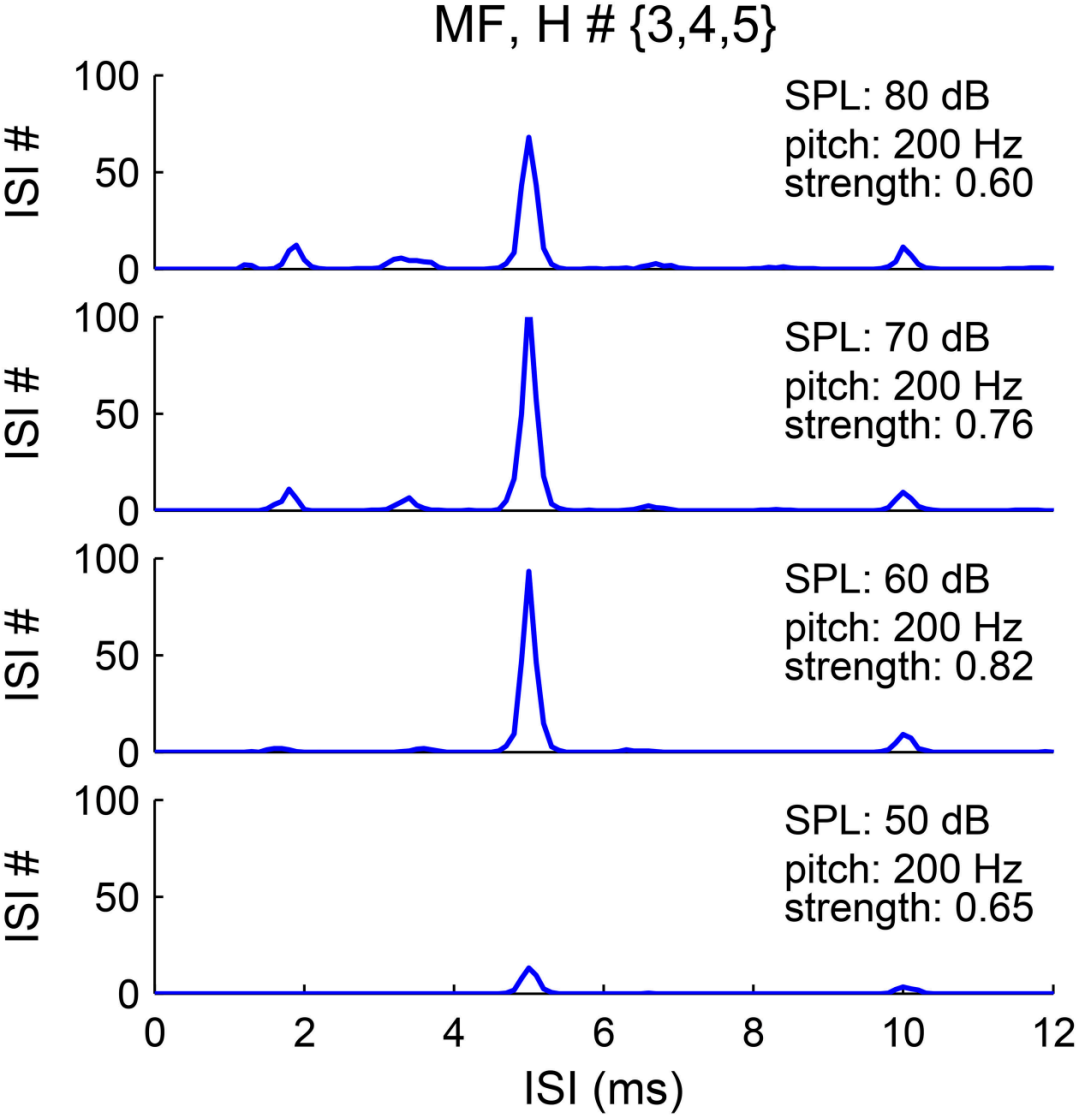
**Pitch frequency estimates are invariant to sound level**. The pooled first-order ISI histograms have a major peak at 5 ms (1/F0) for sound level from 50 to 80 dB (bottom to top). The harmonic frequencies of the MF complex are {600, 800, 1000} Hz with fundamental frequency F0 = 200 Hz. The lower amplitude of the peak at sound level 50 dB (bottom) is due to the lower input rate from AN fibers.

The pitch representation in the first-order ISI histograms of the SD units is robust to sound level, although that of AN responses is sensitive to sound level (Cariani and Delgutte, 1996a; Figure 9). The pooled ISI histograms of SDs at different sound levels invariably show a major peak at 1/F0 (Figure 10). A first-order interval histogram is generally sensitive to firing rate due to added spikes, while an all-order interval histogram is more robust. However, since the activated *I*_*KLT*_ suppresses further spikes immediately after an action potential (see Methods), the SD units typically fire only once in each pitch-related period. Besides, the threshold for the SD units to phase-lock at high frequency increases dramatically (Meng et al., 2012, see Figure 5A). Therefore, fewer SD units have additional spikes within a pitch-related period for stimuli of higher pitch.

The model has a narrower frequency range of pitch representation than AC models. The range of frequency representation depends on the phase-locking capability of the SD units and the synaptic strengths of the AN inputs. Stronger synapses from AN to SD (larger *g*_*E*_ in Equation 6) can extend the tuning curve to higher pitch frequencies for both pure tones and the MF complexes (Supplementary Figure 1A). However, frequency doubling may occur for low F0 when synapses are too strong such that the SD units respond to the other coincidences within a cycle and shorter ISIs become more prevalent (Supplementary Figures 1A,B). A gradient of increasing synaptic strengths of AN inputs with increasing BF can help better drive the SD units for high F0 tones while maintaining to moderate levels the drive from low F0 tones. Another reason that an SD unit fails to respond to high F0 tones is that the total AN input each SD unit receives are dispersed within a cycle, giving the SD unit less time to recover from *I*_*KLT*_. A tonic inhibition combined with higher input strength can, in principle, serve a modulatory effect on encoding by helping an SD unit to phase-lock to higher frequency. Another possible way to improve entrainment and synchronization is by using a cascade of convergent feedforward SD layers. Although the frequency range is restricted compared to human pitch perception (up to 4 kHz), pitch range of complex tones, such as the MF and amplitude-modulated tones, are generally restricted to low frequencies as well, up to a few hundred hertz (Plack and Oxenham, 2005, Section 3.5.1). Besides, physiological recordings of brain stem neurons also generally show a decrease of entrainment for frequency higher than 500 Hz (Recio-Spinoso, 2012; Joris, 2016). The pitch of a pure tone of higher frequency can be encoded by its spectral content.

### 4.3. Pitch extraction mechanisms

The SD units in our model enhance the pitch representation in the first-order ISIs compared to the AN fibers. Nevertheless, encoding by the model is temporally based. In the current model formulation, we do not model mechanistically how pitch is extracted from a first-order ISI histogram. When entrainment is perfect, the firing rate of an SD unit can be taken as the pitch frequency. In this case, the pitch frequency estimate is a monotonic function of firing rate and the range of pitch frequency representation depends on the phase-locking capability of the neuron. Joris (2016) also propose that entrained phase-locking of neurons in the brain stem can serve as a representation of pitch, as an alternative to the autocorrelations of AN spike times.

Another possible representation of pitch is a rate-place code of band-pass tuning for pitch frequency. An oscillator with a narrowly-tuned resonant frequency for periodic forcing can presumably select the ISI that corresponds to its resonant frequency. The first-order ISI histogram in the third stage of our model can then be transformed into a rate code; neurons that prefer the most frequently occurring ISI fire the most. Modeling of the chopper units of the ventral cochlear nucleus has been suggested to underlie a temporal place code due to the intrinsic oscillations of the chopper units (Wiegrebe and Meddis, 2004; Meddis and O'Mard, 2006). The modulation transfer function of a sustained chopper unit exhibits preferential firing at the unit's chopping rate as well as at the harmonics of its chopping rate. Therefore, a sustained chopper unit can entrain either to the rate of the waveform envelope when it receives unresolved harmonic inputs, or to the fundamental frequency when it receives a resolved component input which is a harmonic of its chopping rate. However, such models with chopper units are not adequate to quantify a pitch accurately due to the broad modulation transfer functions of chopper units. The pitch is represented implicitly as a profile of firing rates of units across chopping rates. These models cannot replicate the linear relationship between pitch and shift frequency for inharmonic complexes as well as the multiple pitch phenomenon of the IRN stimuli. Moreover, these models are also limited to low pitch frequencies (a few hundred Hz) since the observed band-pass modulation transfer functions of the sustained chopper units as well as of the inferior colliculus neurons are peaked at low frequencies (Hewitt and Meddis, 1994). A biophysical implementation of a narrowly tuned oscillator is a future direction.

On the other hand, since most psychophysical experiments of pitch involve pitch-matching rather than pitch identification, it is not necessary to derive an exact pitch value. It is possible that the brain uses the temporal information directly to compare pitch representations of two successive tone stimuli, rather than transforming to a rate code for an exact value of pitch. The regular firing pattern of the SD units in our model may be stored in working memory and utilized later to compare with the temporal pattern invoked by the second matching stimuli.

## 5. Conclusions

We developed a computational model for pitch frequency estimation with biophysically-based slope-detector neurons. The slope detectors receive AN inputs from a broad range of frequency channels and phase-lock at the pitch-related periodicity by detecting coincidence among the convergent AN inputs. The pitch representation is, therefore, greatly enhanced in the first-order inter-spike intervals of the slope-detectors compared to that of the AN fibers. The regular firing pattern of the slope-detectors, invariant with respect to stimulus type, can serve as a neural representation of pitch that may be further transformed into a rate code or utilized directly for pitch discrimination.

## Author contributions

Designed and formulated the model: CH, JR. Implemented and tested the model: CH. Wrote the paper: CH. Edited the manuscript: CH, JR.

## Funding

This work is supported by NIH K18-DC011602 to JR.

### Conflict of interest statement

The authors declare that the research was conducted in the absence of any commercial or financial relationships that could be construed as a potential conflict of interest.
