# Supplementary material for: A Neuronal Network Model for Pitch Selectivity and Representation
Source: Front Comput Neurosci. 2016 Jun 16;10:57. doi: 10.3389/fncom.2016.00057 (PMC4910526; doi:10.3389/fncom.2016.00057)
Supplement: Supplementary file 1 [file Presentation1.PDF]

Supplementary Material: A neuronal network model for pitch selectivity and representation

Chengcheng Huang [1,2*] **and John Rinzel** [1,3]

[1] *Courant Institute of Mathematical Sciences, New York University, New York, NY, USA*
[2] *Department of Mathematics, University of Pittsburgh, Pittsburgh, PA, USA*
[3] *Center for Neural Science, New York University, New York, NY, USA*

Correspondence*:
Chengcheng Huang
Department of Mathematics, University of Pittsburgh, 504 Thackeray Hall, Pittsburgh, PA 15260, chengchenghuang11@gmail.com

1 SUPPLEMENTARY DATA

Some examples of the sound stimuli used in this paper are provided. More sound demos of pitch can be found at `https://auditoryneuroscience.com/pitch`.

Audio 1: A missing fundamental harmonic complex frequency components {600, 800, 1000} Hz (F0=200 Hz).
Audio 2: An inharmonic complex with spacing F0=200 Hz (harmonic number 2-7) and shift 40 Hz.
Audio 3: An inharmonic complex with spacing F0=200 Hz (harmonic number 2-7) and shift -40 Hz.
Audio 4: A harmonic complex with alternating phase (F0=125 Hz) filtered in the frequency range [125, 625] Hz.
Audio 5: A harmonic complex with sine phase (F0=125 Hz) filtered in the frequency range [125, 625] Hz.
Audio 6: A harmonic complex with alternating phase (F0=125 Hz) filtered in the frequency range [3900-5400] Hz.
Audio 7: A harmonic complex with sine phase (F0=125 Hz) filtered in the frequency range [3900-5400] Hz.
Audio 8: A harmonic complex with a negative Schroeder phase and F0=100 Hz.
Audio 9: A harmonic complex with a positive Schroeder phase and F0=100 Hz.
Audio 10: A harmonic complex with a random phase and F0=200 Hz (frequency components {600, 800, 1000} Hz).
Audio 11: Iterated ripple noise with delay $d = 4$ ms, gain $g = 1$ and iteration number $n = 2$.
Audio 12: Iterated ripple noise with delay $d = 4$ ms, gain $g = -1$ and iteration number $n = 2$.
Audio 13: Iterated ripple noise with delay $d = 4$ ms, gain $g = 1$ and iteration number $n = 8$.
Audio 14: Iterated ripple noise with delay $d = 4$ ms, gain $g = -1$ and iteration number $n = 8$.

2 SUPPLEMENTARY FIGURE

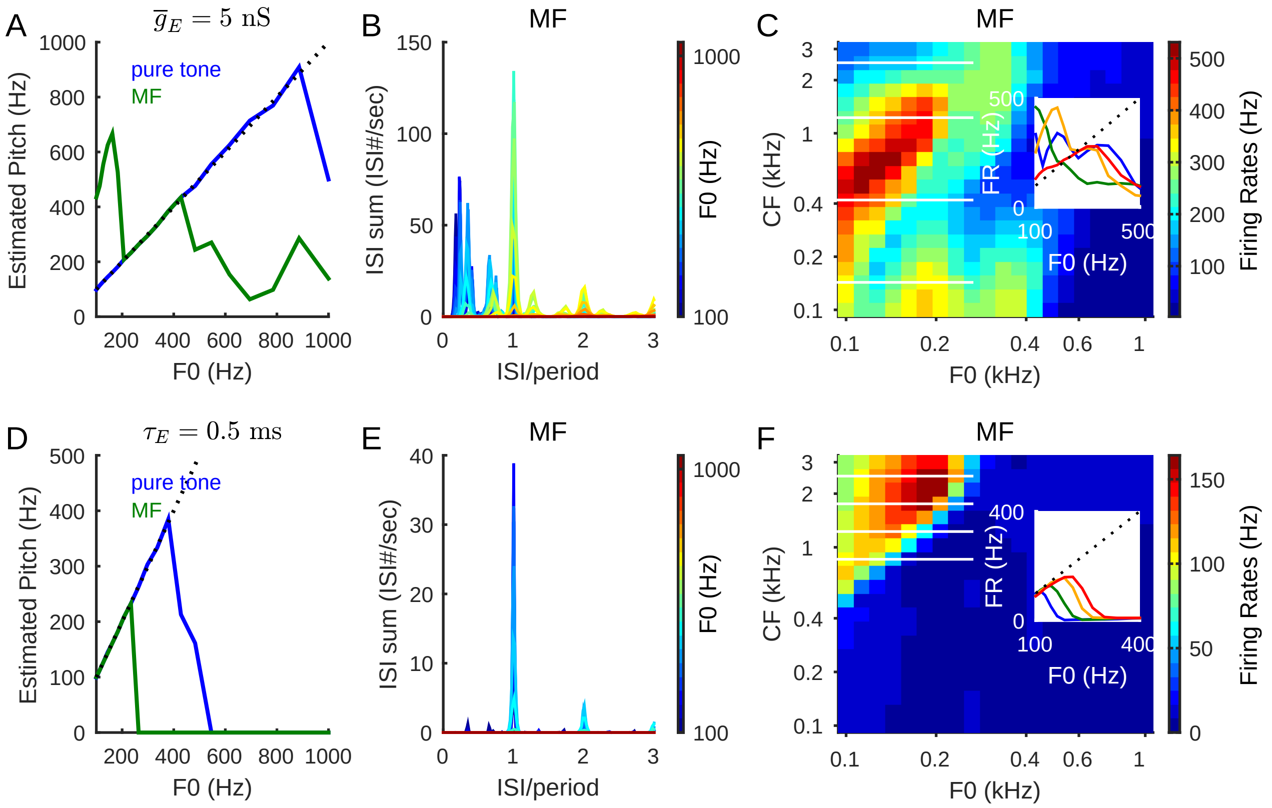

Supplementary Figure 1. Model responses to MF complexes and pure tones with larger synaptic strength or slower synapses, upper and lower panels, respectively. Figure formats are similar to Fig. 4 in the main text. Harmonic numbers for MF complexes are {3, 4, 5} with fundamental frequency, F0, varying logarithmically from 100 to 1000 Hz. For pure tones (in A and D only), F0 refers to tone frequency. (A-C) Synaptic strength from AN to SD is $\bar{g}_E = 5$ nS and the synaptic time constant $\tau_E = 0.07$ ms (Eq. 6). (A) For pure tones, estimated pitch is identical to tone frequency up to about 900 Hz (blue), compared to 500 Hz with weaker synaptic strength (Fig. 4A blue, $\bar{g}_E = 1.5$ nS). For MF complexes, estimated pitch is equal to F0 in the range of 200 – 400 Hz (green), compared to the range 100 – 300 Hz with weaker synaptic strength (Fig. 4A blue). Frequency doubling happens for lower F0, resulting in shorter ISIs and higher estimated pitch. Dotted black line represents equality with F0. (B) The summed ISI histograms of SD units over CF in response to MF complexes of different F0 (color coded). The $x-$axis is ISI normalized by the period (1/F0) of the MF complexes. For MF complexes of low F0, ISIs shorter than a period are more prominent. (C) Firing rates of SD units across CF in response to MF complexes as a function of F0. The SD units at intermediate CFs fire at a higher rate than F0, which results in ISIs smaller than the period 1/F0. These shorter ISIs lead to the overestimate of pitch for low F0 seen in panel A. The $x-$ and $y-$ axes are on a logrithmic scale. Inset: representative firing rate tuning curves as a function of F0 for SD units of CFs 143 Hz (blue), 419 Hz (green), 1226 Hz (orange), 2508 Hz (red). Only for SD units of high CF (e.g. red) do we see entrainment (firing rate along the diagonal line (dotted)). The positions of these four CFs are shown as white horizontal lines in the heat plot. (D-F) Same as A-C with slower synapses, $\tau_E = 0.5$ ms and $\bar{g}_E = 1.5$ nS. (D) The model estimates pitch correctly for a slightly limited range, up to about 400 Hz for pure tones and 250 Hz for MF complexes, compared to that with faster synapses (Fig. 4A, $\tau_E = 0.07$ ms, up to about 500 Hz for pure tones and 300 Hz for MF complexes). (E) The summed ISI histograms of SD units over CF show a narrow dominant peak at the period 1/F0 for F0 up to 250 Hz. (F) The pitch is extracted by SD units from high CF sites, where the AN inputs are modulated by the sound's envelope. The slower synapses degrade the high frequency information of individual harmonics in low frequency channels. Inset: representative firing rate tuning curves as a function of F0 for SD units of CFs 857 Hz (blue), 1226 Hz (green), 1753 Hz (orange), 2508 Hz (red). Responses entrain one to one to modulation of AN inputs before falling off at higher F0. Results are averaged over 50 runs with independent AN input. Pitch and pitch strength are set to zero when the mean firing rate of all SD units is below 15 Hz.
