# Supplementary figures and images for: A Neuronal Network Model for Pitch Selectivity and Representation
Source: Front Comput Neurosci. 2016 Jun 16;10:57. doi: 10.3389/fncom.2016.00057 (PMC4910526; doi:10.3389/fncom.2016.00057)

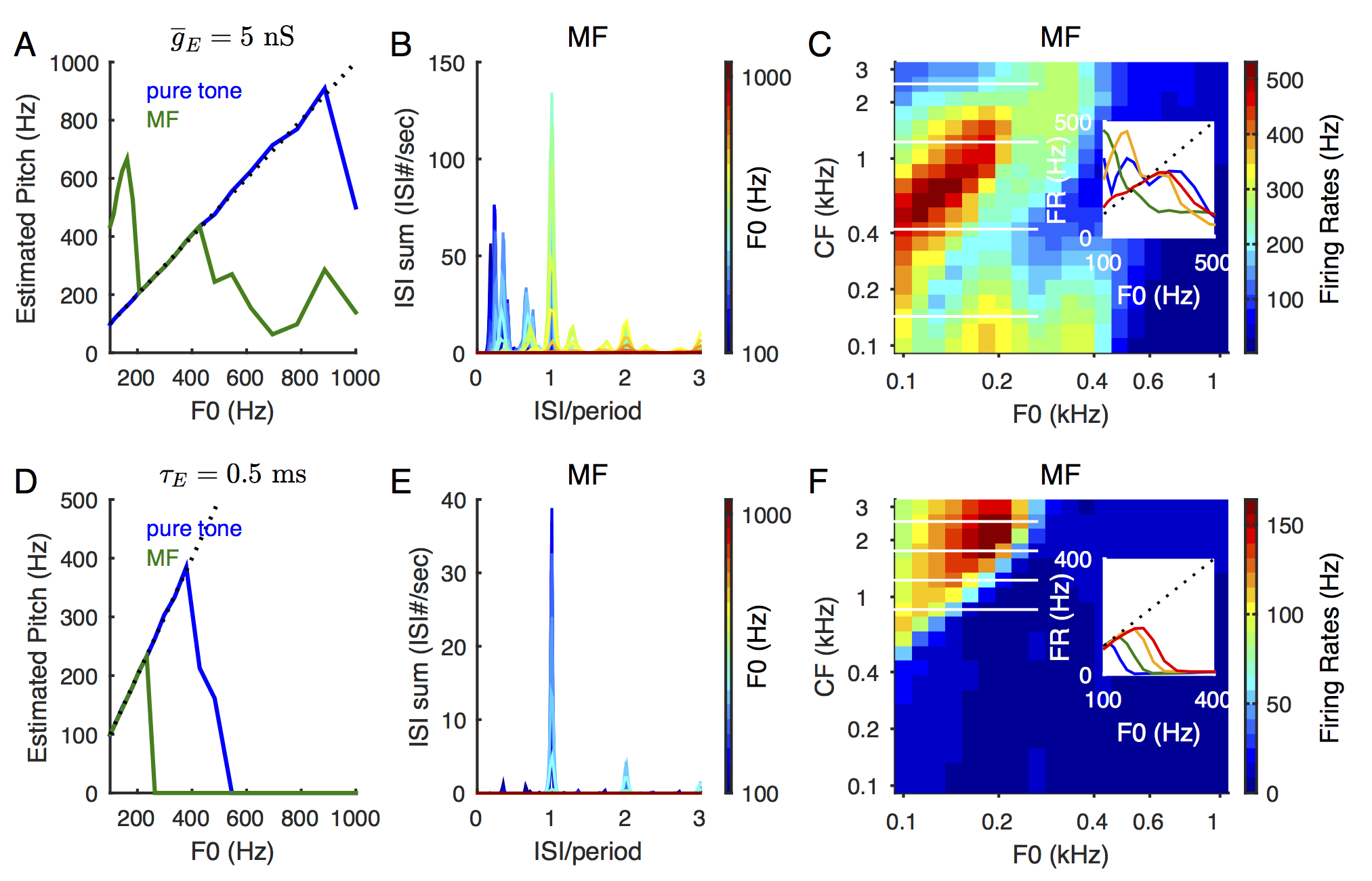

Supplement: Supplementary file 16 [file Image1.TIFF]
